# Analyzing of optimal classifier selection for EEG signals of depression patients based on intelligent fuzzy decision support systems

**DOI:** 10.1038/s41598-023-36095-3

**Published:** 2023-07-14

**Authors:** Saleem Abdullah, Shougi S. Abosuliman

**Affiliations:** 1 Department of Mathematics, Abdul Wali Khan University Mardan, Mardan, KP Pakistan; 2 Department of Port and Maritime Transport, Faculty of Maritime Studies, King Abdulaziz University, Jeddah, 21588 Saudi Arabia

**Keywords:** Neurological disorders, Materials science, Mathematics and computing

## Abstract

Electroencephalograms (EEG) is used to assess patients' clinical records of depression (EEG). The disorder of human thinking is a very complex problem caused by heavy-duty in daily life. We need some future and optimal classifier selection by using different techniques for depression data extraction using EEG. Intelligent decision support is a decision-making process that is automated based on some input information. The primary goal of this proposed work is to create an artificial intelligence-based fuzzy decision support system (AI-FDSS). Based on the given criteria, the AI-FDSS is considered for classifier selection for EEG under depression information. The proposed intelligent decision technique examines classifier alternatives such as Gaussian mixture models (GMM), k-nearest neighbor algorithm (k-NN), Decision tree (DT), Nave Bayes classification (NBC), and Probabilistic neural network (PNN). For analyzing optimal classifiers selection for EEG in depression patients, the proposed technique is criterion-based. First, we develop a general algorithm for intelligent decision systems based on non-linear Diophantine fuzzy numbers to examine the classifier selection technique using various criteria. We use classifier methods to obtain data from depression patients in normal and abnormal situations based on the given criteria. The proposed technique is criterion-based for analyzing optimal classifier selection for EEG in patients suffering from depression. The proposed model for analyzing classifier selection in EEG is compared to existing models.

## Introduction

The significance of multi-criteria decision-making (MCDM) is increasing in tandem with the world's rapid population growth and the resulting problems in medical decision-making due to increasing uncertainty and ambiguity in real-world data. Because traditional tools did not address these types of data uncertainty and ambiguity, decision-makers and medical specialists are turning to modern tools to reduce uncertainty in real-world data. As a result, in 1965^1^, Zadeh proposed the concept of a fuzzy set (FS), which took into account the membership degree (MD) of an element in a set. The membership degree in a fuzzy set did not cover an object's uncertainty. When he added the degree of non-membership (DNM) to an FS and created the so-called intuitionistic fuzzy set (IFS), he was attempting to develop and establish the generalized notion^2^. In 1986, the basic features of the intuitionistic fuzzy set (IFS) were developed and researched. There are some constraints on DM and DNM in the intuitionistic fuzzy set. In some cases, the IFS was unable to explain the uncertainty of real-world data due to IFS constraints. Because the sum of the DM and DNM values is greater than one, the IFS theory has limitations in transmitting information in the DM and DNM of an object. As a result, Yager developed the concept of a Pythagorean fuzzy set (PyFS) in 2013 to address the limitations of IFS theory in real-world applications^3^. PyFS increased the space of DM and DNM to the sum of their squares. The IFS is a special case of a PyFS, implying that PyFS is a more advanced version of an IFS that is more useful to decision-makers. Many indeterminacy and complicated challenges in decision-making applications have been considered by PyFS^4–15^. If an expert assigns an element DM = 0.6 and DNM = 0.65, the given information does not meet the IFS criterion, but it can be clearly expressed using PyFS alone. As a result, in real life, IF decision-making differs from PyF decision-making. As a result, the PyFS has greater authority and form strategic alliances than the IFS.

The IFS and PyFS do not always meet the DM and DNM requirements of an object in decision-making due to some restrictions on DM and DNM. As a result, Yager^16^ developed a new concept called q-rung orthopair fuzzy sets (q-ROFS), which is more general than IFS and PyFS. Yager expanded the space of DM and DNM by increasing the values of DM and DNM in q-ROFS. The DM and DNM square requirements were expanded to include the sum of their powers, i.e. ${{\Upsilon }_{K}}^{q}+{{\Sigma }_{K}}^{q}\le 1$, where $q\ge 1$. When $q=1$ is chosen, q-ROFS is reduced to IFS, and when $q=2$ is chosen, q-ROFS is reduced to PyFS. This means that q-ROFS is more effective and informative than IFS and PyFS in describing uncertainty and ambiguity. Liu and Wang created algebraic operational laws for q-ROFS as well as a decision-making method (DMM) for q-ROFS^17^. The McLaurin symmetric mean aggregation operators for q-ROFS are discussed in^18^. Partitioned Bonferroni mean aggregation operators^19^, Heronian mean aggregation operators^20^, and power Bonferroni mean aggregation operators^21^ are some useful aggregation operators for q-ROFS developed by various authors. Raiz and Hashmi^22^ proposed and developed the notion of the linear Diophantine fuzzy set (LDFS) by including reference parameters in FS, IFS, PyFS, and q-ROFS, as well as relaxing the restriction on DM and DNM in FS, IFS, PyFS, and q-ROFS, respectively. LDFS has a larger space than IFS, PyFS, and q-ROFS and is also useful for decision-making problems^23^. The LDFS is a new and better concept than the previous extension of FS to account for uncertainty and ambiguity in real-life situations. As a result, various authors began working on it. The authors extended the LDFS concept to the concepts of rough soft set and rough soft LDFS, which they applied to the selection of sustainable materials^23^. Ayub et al.^24^ defined algebraic properties and their relationships for the LDFS. Lampan et al.^25^ developed LDF Einstein's aggregation to solve decision-making problems in LDFS settings. In^26^, Kamaci extended the LDFS concept to algebraic structures and produced a large number of algebraic structures under LDFS. The authors of^27^ created a new concept called q-rung linear Diophantine fuzzy set (q-RLDFS) and applied it to COVID19. The above-detailed discussion of AOs for different structures of FS has been discussed and these AOs developed based on T-norms (TNs) and T-conorms (TCNS). The TNs has used to aggregate two FS, IFS, PyFS, and q-ROFS to a single while copulas are described with the probabilistic approach in fuzzy environments. The copulas and Co-copulas are well known as TNs and TCNS, respectively. The copulas have two different features one is copula and another is Co-copulas and these copulas are flexible in the decision-making process. Further many researchers extended copulas and Co-copulas to other fields and applied them successfully^28–32^.

Nowadays, a human mental disorder or depression is a major ill in the world. Psychological illness has various characteristics, one of which is an important and consistent low mood. Mental disorder or depression is increasing due to a highly busy life. Depression is a disorder that is affecting more and more people and Persistent depression, depression and frustration are major medical manifestations, in which suicidal tendencies appear in severe cases. At the end of 2020, the World Health Organization (WHO) reported that 350 million people were affected by depression and that it was the second largest disease in the world after heart disease^33,34^. In the middle of the 19th Century, depression properly has studied as a mental disorder disease. Physicians use to diagnose this disease by using different ways of diagnostic and ordinary tests, which these different tests give different results of diagnostic. This concludes that depressed patients with outward signs of trust in self-care to implement a management plan of treatment, and there is no such physical signal to apply as a measurable constraint. This is very hard to succeed in an initial checkup, diagnosis, and anticipation of depression. With the rapid growth of technology of cognitive science and sensor technology in recent years, researchers can apply electroencephalogram (EEG) to record brain activities as physiological signals. The clinical record of disorders is measured by Electroephalogrphy (EEG). Depression and brain movement have a significant connection, which may be measured and recorded using EEG physiological signals. EEG recordings can be used in a variety of clinical brain function tests. EEG signals are used to examine brain activity, according to a large quantity of research^33,35–38^. There are different EEG classifiers to detect the brain activity of depressed patients and the mental disorderliness of depression. Jareda et al.^39^ proposed a new EEG based method for seizure classification based on brain activities to decompose EEG signal. Kukker and Sharma proposed^40^ a novel online Genetic Algorithm assisted Fuzzy Q-Learning and Fuzzy Q-Learning classifiers for epileptic seizures. The visual analysis of common neurological disorders such as epileptic seizures in electroencephalography (EEG) is an oversensitive operation and prone to errors. Therefore Slimen et al.^41^ proposed a robust automatic seizure detection method that can establish a veritable diagnosis of these diseases.

In this work, we develop a medical decision support model to analyze the different EEG classifiers based on five types of entropies under the linear Diophantine fuzzy numbers.

The final step of the decision process is to finalize the decision for the optimal selection of alternatives. This paper deal with the following main objectives:

1. The basic set-theoretical operations of LDFNS based on extended copulas and copulas are considered and the basic operations are enhanced from T-norm and T-conom to extended copulas and copulas for LDFNS.
2. Further the aggregation operators for LDFNS based on the proposed extended operational laws and define linear Diophantine fuzzy Copulas weighted aggregation operators
3. The entropy measure and distance measures develop to determine the weight vector for decision-makers as well as for criteria
4. The Extended TOPSIS method considered under the LDFNS with unknown weight information
5. We develop artificial intelligence-based fuzzy decision support systems (AI-FDSS) and intelligent decision support is an automatic decision process based on some input information.
6. The AI-FDSS is considered for classifier selection for EEG under depression information based on given criteria.

### Motivation of the study

In a group decision or multi-expert decision-making (MEDM) problem, the decision expert needs an operator to aggregate the multi-expert opinion into single and collective decision information. Liu et. al.^35^ defined some different aggregation operators for linguistic Intuitionistic cubic fuzzy sets. The AOs using sine operation has developed by Abdullah et. al.^36^. In^37^, Naeem et.al. developed some similarity measures on fractional orthopair fuzzy sets.

According to the literature, LDFNS is an effective tool for complex decision-making, and it covered the shortcoming of the previous concept of IFS, PFS, q-ROFS. Some addition to the research has been deliberated since its inception, as have several techniques for advancing and enhancing LDFNS with information measures and Aggregation Operators (AOs).

- As compared with previous concepts, the LDFS-based decision-making model is more flexible and reliable with human evaluation information by expanding the space of the degree of membership and degree of non-membership. As a result, it is critical to pay more devotion to Multi-expert decision-making under the LDFNS.
- Finding unknown weight vectors for decision-makers or criteria is a critical issue. To address this issue, the Entropy measure of the LDFS-based procedure to obtain the weight of DM and criteria to avoid the unfavorable influence of weights is used.
- The interdependence between input data is too much for LDFS aggregation operators based on t-norm and t-conorm to handle. Aggregation operators based on Extended-Copulas and Co-copulas for LDFNs are introduced to overcome the limitation of previously developed for LDFNs.
- There are several methods for finding unknown objective weight vectors for decision experts and weight of criteria under LDFNs. We must use the entropy measure to obtain the unknown weight of the decision maker as well as the weight of the criteria.
- The TOPSIS approach paved the way for a more straightforward and usable way to rank alternatives. As a result, when combined with LDFNs in an MCDM setting, the TOPSIS will be a strong authoritative decision-making method other than methods.
- There are different EEG classifiers to detect the brain activity of depressed patients and the mental disorderliness of depression. Several criteria must be taken when selecting the best optimal classifier selection for EEG signals of depression patients. To solve these problems, we must use classifier selection for EEG signals of depression patients

### Contribution of the study

In light of the above literature review, this concludes that there is no detail and implementations of LDFS based on extended copula t-norm and t co-norm to medical decision support models for selecting optimal EEG Classifier for depression possible outcomes. So the goals of this research are to extend LDFS with copula and derived several aggregation operators based on extended copula norms to tackle ambiguity and uncertainty with LDFS settings. There are three main steps in MADM, in which the decision process find the optimal selection of alternative. The MADM process begins with the structure of the decision model, which is used to formulate data information for each alternative based on defined criteria by each decision expert. The second step then begins with the data information of all alternatives based on defined criteria represented by each decision expert's decision matrix and this decision will be normalized if needed. The final step of the decision process is to finalize the decision for the optimal selection of alternatives. The main objectives of this paper are as follows:

1. The basic set-theoretical operations of LDFNS based on extended copulas and copulas are considered and the basic operations are enhanced from T-norm and T-conom to extended copulas and copulas for LDFNS.
2. Further, the aggregation operators for LDFNS based on the proposed extended operational laws and define linear Diophantine fuzzy Copulas weighted aggregation operators
3. The entropy measure and distance measures develop to determine the weight vector for decision-makers as well as for criteria
4. The Extended TOPSIS method considered under the LDFNS with unknown weight information
5. We develop artificial intelligence-based fuzzy decision support systems (AI-FDSS) and intelligent decision support is an automatic decision process based on some input information.
6. The AI-FDSS is considered for classifier selection for EEG under depression information based on given criteria.

## Basic concepts

This section provides the basic terminology and concept of IFS, PyFS, q-ROFS, and their relationship.

### Definition 1

^2^ The IFS was developed by Atanassov in 1986 and he added a degree of non-membership mapping to FS. The intuitionistic fuzzy set has more informative than FS. Now we defined the IFS for a non-empty set. Let's consider a universal set $\wp$. Then, an intuitionistic fuzzy set $\mathcal{L}$ of $\wp$ can be defined as:

$$\mathcal{L}=\left\{\left(\rho , {\varphi }_{\mathcal{L}}\left(\rho \right), { \psi }_{\mathcal{L}}(\rho )\right):\rho \in \wp \right\},$$

where ${\varphi }_{\mathcal{L}}:\rho \to [{0,1}] \& { \psi }_{\mathcal{L}}:\rho \to \left[{0,1}\right]$ are mappings of membership and non-membership, respectively subject to condition $0\le {\varphi }_{\mathcal{L}}\left(\rho \right)+{ \psi }_{\mathcal{L}}(\rho )\le 1$. The degree of membership (DM) is denoted by ${\varphi }_{\mathcal{L}}\left(\rho \right)$ and the degree of non-membership (DNM) is represented by ${\psi }_{\mathcal{L}}(\rho )$ of $\rho \in \wp$.

### Definition 2

^3^ The generalization of IFS is defined by Yager and he expands the space of membership and non-membership. Consider $\wp$ be a universal set. Then the Pythagorean fuzzy set (PyFS) $\mathcal{L}$ of $\wp$ can be defined as:

$$\mathcal{L}=\left\{\left(\rho , {\varphi }_{\mathcal{L}}\left(\rho \right), { \psi }_{\mathcal{L}}(\rho )\right) : \rho \in \wp \right\},$$

where the mapping of membership and non-membership is represented by ${\varphi }_{\mathcal{L}}:\rho \to [{0,1}] \& { \psi }_{\mathcal{L}}:\rho \to \left[{0,1}\right]$. Also subject to the condition 0 ≤${\left({\varphi }_{\mathcal{L}}\left(\rho \right) \right)}^{2}+{\left({ \psi }_{\mathcal{L}}\left(\rho \right)\right)}^{2}$  ≤ 1,$\forall \rho \in \wp$. The (DM) is denoted by ${\varphi }_{\mathcal{L}}\left(\rho \right)$, and the DNM is represented by ${\psi }_{\mathcal{L}}(\rho )$ of $\rho \in \wp$.

### Definition 3

^16^ Let's take a universal set $\wp$. Then, the q-rung orthopair FS (q-ROFS) $\mathcal{L}$ of $\wp$ can be defined as:

$$\mathcal{L}=\left\{\left(\rho , {\varphi }_{\mathcal{L}}\left(\rho \right), { \psi }_{\mathcal{L}}(\rho )\right) : \rho \in \wp \right\}$$

where the mapping of membership and non-membership is represented by ${\varphi }_{\mathcal{L}}:\rho \to [{0,1}] \& { \psi }_{\mathcal{L}}:\rho \to \left[{0,1}\right],$ respectively. Also subject to the condition 0 ≤${\left({\varphi }_{\mathcal{L}}\left(\rho \right) \right)}^{q}+{\left({ \psi }_{\mathcal{L}}\left(\rho \right)\right)}^{q}$  ≤ 1 with $\mathcal{q}>2.$, $\forall \rho \in \wp$. The (DM) is denoted by ${\varphi }_{\mathcal{L}}\left(\rho \right)$, and the DNM is represented by ${\psi }_{\mathcal{L}}(\rho )$ of $\rho \in \wp$.

Raiz and Hashmi decided to expand the LDFS concept by including reference parameters. LDFS has a larger space than IFS, PyFS, and q-ROFS and is also useful for decision-making problems^23^. The LDFS is a new and superior concept to the previous extension of FS to conceal ambiguity and uncertainty in real-world situations. As a result, LDFS was created to reduce ambiguity and uncertainty in real-world problems.

### Definition 4

^23^ Let's consider a universal set $\wp$. Then, the LDFS is denoted by $\mathcal{L}$ of $\wp$ and is defined by the following form;

$$\mathcal{L}=\left\{\langle \rho ,\left({\varphi }_{\mathcal{L}}\left(\rho \right),{ \psi }_{\mathcal{L}}(\rho )\right), \left(\varrho ,\sigma \right)\rangle :\rho \in \mathrm{\wp }\right\},$$

where DM is signified by ${\varphi }_{\mathcal{L}}\left(\rho \right)$ and DNM is signified by ${\psi }_{\mathcal{L}}\left(\rho \right),$ respectively and the reference parameters are denoted by $\left(\varrho ,\sigma \right)$ with subject to the following conditions;

$${\varphi }_{\mathcal{L}}\left(\rho \right)\cdot {\varrho }^{q}+{ \psi }_{\mathcal{L}}\left(\rho \right){\sigma }^{q}\le 1 and {\varrho }^{q}+{\sigma }^{q}\le 1$$

### Definition 5

^23^ Consider $\mathcal{L}, {\mathcal{L}}_{1}, {\mathcal{L}}_{2}$ be three LDFS. Then some basic operations can be defined as:

1. $${\mathcal{L}}_{1}\oplus {\mathcal{L}}_{2}=\left\{\left({\varphi }_{{\mathcal{L}}_{1}}\left(\rho \right)+{\varphi }_{{\mathcal{L}}_{2}}\left(\rho \right)-{\varphi }_{{\mathcal{L}}_{1}}\left(\rho \right){\varphi }_{{\mathcal{L}}_{2}}\left(\rho \right)\right),\left({\psi }_{{\mathcal{L}}_{1}}\left(\rho \right){\psi }_{{\mathcal{L}}_{2}}\left(\rho \right)\right),\left(\left({\varrho }_{1}+{\varrho }_{2}-{\varrho }_{1}{\varrho }_{2}\right),{\sigma }_{1}{\sigma }_{2}\right)\right\},$$
2. $${\mathcal{L}}_{1}\otimes {\mathcal{L}}_{2}=\left\{\left(\left({\varphi }_{{\mathcal{L}}_{1}}\left(\rho \right){\varphi }_{{\mathcal{L}}_{2}}\left(\rho \right)\right),\left({\psi }_{{\mathcal{L}}_{1}}\left(\rho \right)+{\psi }_{{\mathcal{L}}_{2}}\left(\rho \right)-{\psi }_{{\mathcal{L}}_{1}}\left(\rho \right){\psi }_{{\mathcal{L}}_{2}}\left(\rho \right)\right)\right),\left({\varrho }_{1}{\varrho }_{2},\left({\sigma }_{1}+{\sigma }_{2}-{\sigma }_{1}{\sigma }_{2}\right),\right)\right\},$$
3. $$\gamma {\mathcal{L}}_{1}=\left\{\left(1-{\left(1-\left({\varphi }_{\mathcal{L}}\left(\rho \right)\right)\right)}^{ \gamma },{\left({\psi }_{\mathcal{L}}\left(\rho \right)\right)}^{\gamma }\right),\left(1-{\left(1-\left(\varrho \right)\right)}^{ \gamma },{\left(\sigma \right)}^{\gamma }\right)\right\}$$,
4. $${\mathcal{L}}_{1}^{\gamma }=\left\{\left({\left({\varphi }_{\mathcal{L}}\left(\rho \right)\right)}^{\gamma },1-{\left(1-\left({\psi }_{\mathcal{L}}\left(\rho \right)\right)\right)}^{ \gamma }\right),\left(1-{\left(1-\left(\varrho \right)\right)}^{ \gamma },{\left(\sigma \right)}^{\gamma }\right)\right\}.$$

### Definition 6

^32^ A function of form two-dimensional function ${\complement }^{^\circ }:{[{0,1}]}^{2} \to [{0,1}]$ is called Copula subject to if ${\complement }^{^\circ }$ holds boundary condition and monotonicity condition i.e.

$$(1) {\complement }^{^\circ }\left(x,1\right)={\complement }^{^\circ }\left(1,x\right)=x, {\complement }^{^\circ }\left(x,0\right)={\complement }^{^\circ }\left({1,0}\right)=0$$

$$(2) {\complement }^{^\circ }\left({x}_{1},{y}_{1}\right)-{\complement }^{^\circ }\left({x}_{2},{y}_{1}\right)- {\complement }^{^\circ }\left({x}_{1},{y}_{2}\right)+{\complement }^{^\circ }\left({x}_{2},{y}_{2}\right)\ge 0$$,where $$x,{x}_{j}, {y}_{j}\in \left[{0,1}\right] , j={1,2}$$ and $${x}_{1}\le {x}_{2}$$, $${y}_{1}\le {y}_{2}$$.

### Definition 7

A Archimedean copula^32^ ${\complement }^{^\circ }$ can be defined as ${\complement }^{^\circ }\left({\mu }_{1},{\mu }_{2}\right)= \psi [\phi ({\mu }_{1})+\phi ({\mu }_{2})]$, where $\phi$ is strictly decreasing and continuous function from [0,1] to $[0,+\infty ]$ with subject to $\phi \left(1\right)-0$ and a continuous and strictly decreasing function *φ* from $[{0,1}]$ to $[0,+\infty ]$ with $\phi \left(1\right)=0$ and $\psi$ is a function from $[0,+\infty ]$ to $[{0,1}]$ and mathematically can be defined as;

1 $$\psi (s)=\left\{\begin{array}{l}{\phi }^{-1}\left(s\right), for s\in \left[0,\phi \left(0\right)\right]:\\ 0, for s\in \left[\phi \left(0\right),+\infty \right].\end{array}\right.$$

### Definition 8

In^19^, the author investigated that if copula ${\complement }^{^\circ }$ increasing strictly on ${[{0,1}]}^{2}, { \phi }^{-1}\to [0,+\infty]$ and $\left(0\right)=+\infty$, then Archimedes copulas can be written as ${\complement }^{^\circ }\left({\mu }_{1},{\mu }_{2}\right)= {\phi }^{-1}\left[\phi \left({\mu }_{1}\right)+\phi \left({\mu }_{1}\right)\right].$

The strict generator and copulas are denoted by the function $\phi$ and ${\complement }^{^\circ }$. The Co-copula is a mapping ${\complement }^{^\circ }:{[{0,1}]}^{2} \to [{0,1}]$ and using the copula to define co-copula as ${\complement }^{\star }\left({\mu }_{1},{\mu }_{2}\right)= 1-{\complement }^{^\circ }(1-{\mu }_{1}, 1-{\mu }_{2})$

## Methods

In this section, we generalized the aggregation operators of LDFSs by using the concept of extended copulas and copula. First, we proposed extended copulas and generators of extended copulas for LDFSs.

### Operational laws of LDFSs using extended copulas

This section provides a detailed description of the operational laws of extended copulas^32^ and their generators and also discuss their fundamental properties. These operations shall be used in the development of AOs of LDFSs.

#### Definition 9

Consider ${\mathcal{L}}_{1}=\langle \left({\varphi }_{{\mathcal{L}}_{1}}\left({\rho }_{i}\right),{ \psi }_{{\mathcal{L}}_{1}}({\rho }_{i})\right), \left({\varrho }_{i},{\sigma }_{i}\right)\rangle$ and ${\mathcal{L}}_{2}=\langle \left({\varphi }_{{\mathcal{L}}_{2}}\left({\rho }_{i}\right),{ \psi }_{{\mathcal{L}}_{2}}({\rho }_{i})\right), \left({\varsigma }_{i},{\tau }_{i}\right)\rangle$ are two LDFSs and $\theta >0$. Then, the operational law is defined as

(LD1) $${\mathcal{L}}_{1}\oplus{\mathcal{L}}_{2}=\left\{\left({C}^{*}\langle {\varphi }_{{\mathcal{L}}_{1}},{\varphi }_{{\mathcal{L}}_{2}}\rangle ,C\langle {\varphi }_{{\mathcal{L}}_{1}},{\varphi }_{{\mathcal{L}}_{2}}\rangle \right)\left({C}^{*}\left({\varrho }_{i},{\varsigma }_{i}\right),C\left({\sigma }_{i},{\tau }_{i}\right)\right)\right\}$$,

(LD2) ${\mathcal{L}}_{1}\otimes{\mathcal{L}}_{2}=\left\{\left(C\langle {\varphi }_{{\mathcal{L}}_{1}},{\varphi }_{{\mathcal{L}}_{2}}\rangle ,{C}^{*}\langle {\varphi }_{{\mathcal{L}}_{1}},{\varphi }_{{\mathcal{L}}_{2}}\rangle \right)\left(C\left({\varrho }_{i},{\varsigma }_{i}\right),{C}^{*}\left({\sigma }_{i},{\tau }_{i}\right)\right)\right\}$,where ${C}^{*}\langle x,y\rangle =1-C(1-x,1-y)$ and $C\left(x,y\right)={\vartheta }^{-1}\left(\vartheta \left(x\right), \vartheta (y)\right)$, and $\vartheta \left(x\right)$ can be any function from the Table 1.

**Table 1:** The $\vartheta \left(x\right)$ functions and their extended copulas and extended co-copulas.

S. no	$\vartheta \left(x\right)$	$C\left(x,y\right)$	${C}^{*}\langle x,y\rangle$
1	${\left(-\mathrm{ln}(x)\right)}^{\lambda }$	$xy$	$x+y-xy$
2	${\left(x\right)}^{-\lambda }-1$	$\left({\left(x\right)}^{-\lambda }+{\left(y\right)}^{-\lambda }-1\right)$	${\left(1-x\right)}^{-\lambda }+{\left(1-x\right)}^{-\lambda }-xy$
3	$ln\left(\frac{{e}^{-\lambda x}-1}{{e}^{-\lambda }-1}\right)$	$\left(-\frac{1}{\lambda }\right)ln\left(\frac{\left({e}^{-\lambda x}-1\right)\left({e}^{-\lambda x}-1\right)}{{e}^{-\lambda }-1}+1\right)$	$1+\frac{1}{\lambda }ln\left(\frac{\left({e}^{-\lambda x}-1\right)\left({e}^{-\lambda x}-1\right)}{{e}^{-\lambda }-1}+1\right)$
4	$ln\left(\frac{1-\lambda (1-x)}{x}\right)$	$\left(\frac{xy}{\left(1-\lambda \right)\left(1-x\right)\left(1-y\right)}\right)$	$1-\left(\frac{\left(1-x\right)\left(1-y\right)}{\left(1-\lambda xy\right)}\right)$
5	$-ln\left(1-{\left(1-x\right)}^{\lambda }\right)$	$1-{\left({\left(1-x\right)}^{\lambda }+{\left(1-y\right)}^{\lambda }-{\left(1-x\right)}^{\lambda }{\left(1-y\right)}^{\lambda }\right)}^{\frac{1}{\lambda }}$	${\left({\left(x\right)}^{\lambda }+{\left(y\right)}^{\lambda }-{\left(x\right)}^{\lambda }{\left(y\right)}^{\lambda }\right)}^{\frac{1}{\lambda }}$

Using the operation rules 1 and 2 of the Definition (4) to find the nth sum and product in Eqs. (5) and (6).

#### Definition 10

Consider LDFV $\mathcal{L}=\langle \left({\varphi }_{\mathcal{L}},{ \psi }_{\mathcal{L}}\right), \left(\varrho ,\sigma \right)\rangle$ and $n\in {Z}^{+}$. Then, the $n\mathcal{L} = \overbrace {{\mathcal{L} \oplus \mathcal{L} \oplus \mathcal{L} \ldots \mathcal{L}}}^{n}$ is also LDFV and

5 $$n\mathcal{L}=\left\{\left(1-{\vartheta }^{-1}\left(n\vartheta (1-{\varphi }_{\mathcal{L}}\right), {\vartheta }^{-1}\left(n\vartheta ({ \psi }_{\mathcal{L}}\right)\right),\left(1-{\vartheta }^{-1}\left(n\vartheta (1-\varrho \right), {\vartheta }^{-1}\left(n\vartheta (\sigma \right)\right)\right\}$$

6 $${\mathcal{L}}^{n}=\left\{\left({\vartheta }^{-1}\left(n\vartheta ({\varphi }_{\mathcal{L}}\right),1-{\vartheta }^{-1}\left(n\vartheta (1-{ \psi }_{\mathcal{L}}\right)\right),\left({\vartheta }^{-1}\left(n\vartheta (\varrho \right),1-{\vartheta }^{-1}\left(n\vartheta (1-\sigma \right)\right)\right\}$$

***Proof*** To prove the above equation, consider n = 1, then

$$1\mathcal{L}=\left\{\left(1-{\vartheta }^{-1}\left(1\vartheta (1-{\varphi }_{\mathcal{L}}\right), {\vartheta }^{-1}\left(1\vartheta ({ \psi }_{\mathcal{L}}\right)\right),\left(1-{\vartheta }^{-1}\left(1\vartheta (1-\varrho \right), {\vartheta }^{-1}\left(1\vartheta (\sigma \right)\right)\right\}$$

$$=\langle \left({\varphi }_{\mathcal{L}},{ \psi }_{\mathcal{L}}\right), \left(\varrho ,\sigma \right)\rangle$$

Now suppose Eq. (32) holds for $n=k$, then we have

$$k\mathcal{L}=\left\{\left(1-{\vartheta }^{-1}\left(k\vartheta (1-{\varphi }_{\mathcal{L}}\right), {\vartheta }^{-1}\left(k\vartheta ({ \psi }_{\mathcal{L}}\right)\right),\left(1-{\vartheta }^{-1}\left(k\vartheta (1-\varrho \right), {\vartheta }^{-1}\left(k\vartheta (\sigma \right)\right)\right\}$$

Now prove it for $n=k+1$, then,

$$\left(k+1\right)\mathcal{L}=k\mathcal{L}\oplus\mathcal{L}=\left\{\left(\mathrm{K},\Gamma \right), \left(\Delta ,\Theta \right)\right\},$$

where $$\mathrm{K}={C}^{*}\left(1-{\vartheta }^{-1}\left(k\vartheta (1-{\varphi }_{\mathcal{L}}\right),{\varphi }_{\mathcal{L}}\right)=1-C\left(1-\left(1-{\vartheta }^{-1}\left(k\vartheta (1-{\varphi }_{\mathcal{L}}\right)\right),1-{\varphi }_{\mathcal{L}}\right)$$

$$=1-C\left({\vartheta }^{-1}\left(k\vartheta (1-{\varphi }_{\mathcal{L}}\right),1-{\varphi }_{\mathcal{L}}\right)=1-{\vartheta }^{-1}\left(\vartheta \left({\vartheta }^{-1}\left(k\vartheta (1-{\varphi }_{\mathcal{L}}\right)+\vartheta \left(1-{\varphi }_{\mathcal{L}}\right)\right)\right)$$

$$=1-{\vartheta }^{-1}\left(\left(k\vartheta (1-{\varphi }_{\mathcal{L}}\right)+\vartheta \left(1-{\varphi }_{\mathcal{L}}\right)\right)=1-{\vartheta }^{-1}\left(\left(\left(k+1\right)\vartheta (1-{\varphi }_{\mathcal{L}}\right)\right)$$

Similarly, we can calculate the following;

$$\Gamma ={\vartheta }^{-1}\left(\left(\left(k+1\right)\vartheta ({ \psi }_{\mathcal{L}}\right)\right),\Delta =1-{\vartheta }^{-1}\left(\left(\left(k+1\right)\vartheta (1-\varrho \right)\right),\Theta ={\vartheta }^{-1}\left(\left(k+1\right)\vartheta (\sigma \right)$$

Thus,

$$\left(k+1\right)\mathcal{L}=\left\{\begin{array}{c}\left(1-{\vartheta }^{-1}\left(\left(k+1\right)\vartheta (1-{\varphi }_{\mathcal{L}}\right), {\vartheta }^{-1}\left(k\vartheta ({ \psi }_{\mathcal{L}}\right)\right),\\ \left(1-{\vartheta }^{-1}\left(\left(k+1\right)\vartheta (1-\varrho \right), {\vartheta }^{-1}\left(\left(k+1\right)\vartheta (\sigma \right)\right)\end{array}\right\}$$

Hence, this trues for all $n\in {Z}^{+}$.

We know from Eqs. (5) and (6). we have for any α $>0$,

(LD3) $$\mathrm{\alpha }\mathcal{L}=\left\{\left(1-{\vartheta }^{-1}\left(\mathrm{\alpha }\vartheta (1-{\varphi }_{\mathcal{L}}\right), {\vartheta }^{-1}\left(\mathrm{\alpha }\vartheta ({ \psi }_{\mathcal{L}}\right)\right),\left(1-{\vartheta }^{-1}\left(\mathrm{\alpha }\vartheta (1-\varrho \right), {\vartheta }^{-1}\left(\mathrm{\alpha }\vartheta (\sigma \right)\right)\right\}$$

(LD4) $${\mathcal{L}}^{\mathrm{\alpha }}=\left\{\left({\vartheta }^{-1}\left(\mathrm{\alpha }\vartheta ({\varphi }_{\mathcal{L}}\right),1-{\vartheta }^{-1}\left(\mathrm{\alpha }\vartheta (1-{ \psi }_{\mathcal{L}}\right)\right),\left({\vartheta }^{-1}\left(\mathrm{\alpha }\vartheta (\varrho \right),1-{\vartheta }^{-1}\left(\mathrm{\alpha }\vartheta (1-\sigma \right)\right)\right\}$$

### Linear Diophantine fuzzy copulas aggregation operators

The section is related to the detailed description of aggregation operators of LDFVs using the extended copulas and extended co-copulas operations. We propose linear Diophantine fuzzy weighted copulas aggregation operators and their types.

Consider the family of LDFVs $\mathrm{\rm H}=\left\{{\mathcal{L}}_{i}=\left({\varphi }_{\mathcal{L}}\left({\rho }_{i}\right),{ \psi }_{\mathcal{L}}({\rho }_{i})\right), \left({\varrho }_{i},{\sigma }_{i}\right),i=\mathrm{1,2},\dots ,n \right\}.$ Then, the mapping $LDFWCA:{H}^{n}\to H$ is called linear Diophantine fuzzy weight copulas aggregation (LDFWCA) operators and mathematically written as follows:

7 $$LDFWCA\left({\mathcal{L}}_{1},{\mathcal{L}}_{2},{\mathcal{L}}_{3},\dots ,{\mathcal{L}}_{n}\right)={\oplus}_{i=1}^{n}{\omega }_{i}{\mathcal{L}}_{i},$$

where ${\omega }_{i}$ is the weight of each ${\mathcal{L}}_{i}$ such that ${\omega }_{i}\in [\mathrm{0,1}]$ and $\sum_{i=1}^{n}{\omega }_{i}$. If we put ${\omega }_{i}=\frac{1}{n}$, then LDFWCA reduce to LDFCA of dimension n which define as;

8 $$LDFWCA=\left({\mathcal{L}}_{1},{\mathcal{L}}_{2},{\mathcal{L}}_{3},\dots ,{\mathcal{L}}_{n}\right)={\frac{1}{2}\mathcal{L}}_{1}\oplus{\frac{1}{2}\mathcal{L}}_{2}\oplus{\frac{1}{2}\mathcal{L}}_{3}\oplus\dots \oplus{\frac{1}{2}\mathcal{L}}_{1}$$

Now we use the properties (LD1) and (LD3) in Eq. (7), and we get the following LDFWCA operator as following;

9 $$LDFWCA\left({\mathcal{L}}_{1},{\mathcal{L}}_{2},{\mathcal{L}}_{3},\dots ,{\mathcal{L}}_{n}\right)=\left\{\begin{array}{c}\left(1-{\vartheta }^{-1}\left(\sum_{i=1}^{n}{\omega }_{i}\vartheta (1-{\varphi }_{{\mathcal{L}}_{i}})\right), {\vartheta }^{-1}\left(\sum_{i=1}^{n}{\omega }_{i}\vartheta \left({ \psi }_{{\mathcal{L}}_{i}}\right)\right)\right),\\ \left(1-{\vartheta }^{-1}\left(\sum_{i=1}^{n}{\omega }_{i}\vartheta \left(1-{\varrho }_{i}\right)\right), {\vartheta }^{-1}\left(\sum_{i=1}^{n}{\omega }_{i}\vartheta \left({\sigma }_{i}\right)\right)\right)\end{array}\right\}$$

where ${\omega }_{i}$ is the weight of each ${\mathcal{L}}_{i}$ such that ${\omega }_{i}\in [\mathrm{0,1}]$ and $\sum_{i=1}^{n}{\omega }_{i}$.

Now, we will discuss some different types of LDFWCA operators by using the values of $\vartheta (x)$, in Eq. (9).

Case 1. If we use the value of $\vartheta \left(x\right)={\left(-\mathrm{ln}(x)\right)}^{\lambda }$, where ${\vartheta }^{-1}\left(x\right)={e}^{-{x}^{\frac{1}{\lambda }}}$ and $\lambda \ge 1$, then Eq. (9) should be as follows;

10 $$LDFWCA\left({\mathcal{L}}_{1},\dots ,{\mathcal{L}}_{n}\right)=\left\{\begin{array}{c}\left(1-{e}^{{\left(\sum_{i=1}^{n}{\omega }_{i}{\left(-ln(1-{\varphi }_{{\mathcal{L}}_{i}}\right)}^{\lambda }\right)}^{\frac{1}{\lambda }}}, {e}^{{\left(\sum_{i=1}^{n}{\omega }_{i}{\left(-ln({ \psi }_{{\mathcal{L}}_{i}}\right)}^{\lambda }\right)}^{\frac{1}{\lambda }}}\right),\\ \left(1-{e}^{{\left(\sum_{i=1}^{n}{\omega }_{i}{\left(-ln(1-{\varrho }_{i}\right)}^{\lambda }\right)}^{\frac{1}{\lambda }}}, {e}^{{\left(\sum_{i=1}^{n}{\omega }_{i}{\left(-ln({\sigma }_{i}\right)}^{\lambda }\right)}^{\frac{1}{\lambda }}}\right)\end{array}\right\}$$

Case 2. Case 1. If we use the value of $\vartheta \left(x\right)={\left(x\right)}^{-\lambda }-1$, where ${\vartheta }^{-1}\left(x\right)={\left(x+1\right)}^{\frac{1}{\lambda }}$ and $\lambda \ge -1$, then Eq. (9) should be as follows;

11 $$LDFWCA\left({\mathcal{L}}_{1},\dots ,{\mathcal{L}}_{n}\right)=\left\{\begin{array}{c}\left(1-{\left(\sum_{i=1}^{n}{\omega }_{i}\left({\left(1-{\varphi }_{{\mathcal{L}}_{i}}\right)}^{-\lambda }-1\right)+1\right)}^{-\frac{1}{\lambda }},{\left(\sum_{i=1}^{n}{\omega }_{i}\left({\left({ \psi }_{{\mathcal{L}}_{i}}\right)}^{-\lambda }-1\right)+1\right)}^{-\frac{1}{\lambda }}\right),\\ \left(1-{\left(\sum_{i=1}^{n}{\omega }_{i}\left({\left(1-{\varrho }_{i}\right)}^{-\lambda }-1\right)+1\right)}^{-\frac{1}{\lambda }}, {\left(\sum_{i=1}^{n}{\omega }_{i}\left({\left({\sigma }_{i}\right)}^{-\lambda }-1\right)+1\right)}^{-\frac{1}{\lambda }}\right)\end{array}\right\}$$

Case 3. If we use the value of $\vartheta \left(x\right)={\left(x\right)}^{-\lambda }-1$, where ${\vartheta }^{-1}\left(x\right)={\left(x+1\right)}^{\frac{1}{\lambda }}$ and $\lambda \ge -1$, then linear Diophantine fuzzy Copulas weight aggregation (LDFCWA, LDFCGA), and also for cases 4 and 5, we have LDFCWA, and LDFCGA, respectively.

### Linear Diophantine fuzzy entropy measure

To calculate the differences between the two LDF, this segment developed generalized and weighted generalized distance measures incorporating LDF information based on the distance model^1,2^. To measure the fuzziness of LDF, we propose entropy measures for LDF based on the developed distance operators:

#### LDF-distance measures

Suppose $\mathfrak{I}=\{{\mathfrak{I}}_{1},{\mathfrak{I}}_{2},...,{\mathfrak{I}}_{n}\}$ and $\mathcal{K}=\{{\mathcal{K}}_{1},{\mathcal{K}}_{2},...,{\mathcal{K}}_{n}\}\in \mathrm{LDF}(\wp )$ where ${\mathfrak{I}}_{i}=\left\{\left({\varphi }_{{\mathfrak{I}}_{i}}\left(\rho \right), {\varphi }_{{\mathfrak{I}}_{i}}\left(\rho \right)\right)\left({\varrho }_{{\mathfrak{I}}_{i}},{\sigma }_{{\mathfrak{I}}_{i}}\right)\right\}$ and ${\mathcal{K}}_{i}=\left\{\left({\varphi }_{{\mathcal{K}}_{i}}\left(\rho \right), {\varphi }_{{\mathcal{K}}_{i}}\left(\rho \right)\right)\left({\varrho }_{{\mathcal{K}}_{i}},{\sigma }_{{\mathcal{K}}_{i}}\right)\right\}$ $\left(i\in \aleph \right)$. Then, the generalized distance measure (GDM) is described for any $\Upsilon >0$ > 0 **(**$\in {\mathbb{R}}$**)** as

12 $${\mathrm{d}}^{g}\left(\mathfrak{I},\mathcal{K}\right)={\left(\frac{1}{4n}\sum_{i=1}^{n}\left({\left|\left({\varphi }_{{\mathfrak{I}}_{i}}\right)-\left({\varphi }_{{\mathcal{K}}_{i}}\right)\right|}^{\Upsilon }+{\left|\left({\psi }_{{\mathfrak{I}}_{i}}\right)-\left({\psi }_{{\mathcal{K}}_{i}}\right)\right|}^{\Upsilon }+{\left|\left({\varrho }_{{\mathfrak{I}}_{i}}\right)-\left({\varrho }_{{\mathcal{K}}_{i}}\right)\right|}^{\Upsilon }+{\left|\left({\sigma }_{{\mathfrak{I}}_{i}}\right)-\left({\sigma }_{{\mathcal{K}}_{i}}\right)\right|}^{\Upsilon }\right)\right)}^{\frac{1}{\Upsilon }}$$

Suppose $\mathfrak{I}=\{{\mathfrak{I}}_{1},{\mathfrak{I}}_{2},...,{\mathfrak{I}}_{n}\}$ and $\mathcal{K}=\{{\mathcal{K}}_{1},{\mathcal{K}}_{2},...,{\mathcal{K}}_{n}\}\in \mathrm{LDF}(\wp )$ where ${\mathfrak{I}}_{i}=\left\{\left({\varphi }_{{\mathfrak{I}}_{i}}\left(\rho \right), {\varphi }_{{\mathfrak{I}}_{i}}\left(\rho \right)\right)\left({\varrho }_{{\mathfrak{I}}_{i}},{\sigma }_{{\mathfrak{I}}_{i}}\right)\right\}$ and ${\mathcal{K}}_{i}=\left\{\left({\varphi }_{{\mathcal{K}}_{i}}\left(\rho \right), {\varphi }_{{\mathcal{K}}_{i}}\left(\rho \right)\right)\left({\varrho }_{{\mathcal{K}}_{i}},{\sigma }_{{\mathcal{K}}_{i}}\right)\right\}$ $\left(i\in \aleph \right)$. Then, the weighted generalized distance measure (WGDM) is described for any $\Upsilon >0$**(**$\in {\mathbb{R}}$**)** as

13 $${\mathrm{d}}^{wg}\left(\mathfrak{I},\mathcal{K}\right)={\left(\frac{1}{4n}\sum_{i=1}^{n}{w}_{i}\left({\left|\left({\varphi }_{{\mathfrak{I}}_{i}}\right)-\left({\varphi }_{{\mathcal{K}}_{i}}\right)\right|}^{\Upsilon }+{\left|\left({\psi }_{{\mathfrak{I}}_{i}}\right)-\left({\psi }_{{\mathcal{K}}_{i}}\right)\right|}^{\Upsilon }+{\left|\left({\varrho }_{{\mathfrak{I}}_{i}}\right)-\left({\varrho }_{{\mathcal{K}}_{i}}\right)\right|}^{\Upsilon }+{\left|\left({\sigma }_{{\mathfrak{I}}_{i}}\right)-\left({\sigma }_{{\mathcal{K}}_{i}}\right)\right|}^{\Upsilon }\right)\right)}^{\frac{1}{\Upsilon }}$$

where ${w}_{i} \left(i\in \aleph \right)$.) are weight information, such that ${w}_{i}\ge 0$ and $\sum_{i=1}^{n}{w}_{i}=1$.

1. For $\Upsilon =1$, the above Eq. (13) becomes weighted Hamming distance(WHD).
2. For $\Upsilon =1$, the above Eq. (13) becomes weighted Euclidean distance(WED).
3. For $\Upsilon =1$, the above Eq. (13) becomes weighted Chebychev distance(WCD).

Suppose ${\mathcal{L}}_{i}=\left\{\left({\varphi }_{{\mathcal{L}}_{i}}\left(\rho \right), {\varphi }_{{\mathcal{L}}_{i}}\left(\rho \right)\right)\left({\varrho }_{{\mathcal{L}}_{i}},{\sigma }_{{\mathcal{L}}_{i}}\right)\right\}\in \mathrm{LDF}\left(\wp \right).(i=\{\mathrm{1,2}\} \in \aleph$.The GDM Eq. (12) is reduced as follows:

14 $${\mathrm{d}}^{g}\left({\mathcal{L}}_{1},{\mathcal{L}}_{2}\right)={\left(\frac{1}{4}\left({\left|\left({\varphi }_{{\mathcal{L}}_{1}}\right)-\left({\varphi }_{{\mathcal{L}}_{2}}\right)\right|}^{\Upsilon }+{\left|\left({\psi }_{{\mathcal{L}}_{1}}\right)-\left({\psi }_{{\mathcal{L}}_{2}}\right)\right|}^{\Upsilon }+{\left|\left({\varrho }_{{\mathcal{L}}_{1}}\right)-\left({\varrho }_{{\mathcal{L}}_{2}}\right)\right|}^{\Upsilon }+{\left|\left({\sigma }_{{\mathcal{L}}_{1}}\right)-\left({\sigma }_{{\mathcal{L}}_{2}}\right)\right|}^{\Upsilon }\right)\right)}^{\frac{1}{\Upsilon }}$$

For ${\mathcal{L}}_{i}=\left\{\left({\varphi }_{{\mathcal{L}}_{i}}\left(\rho \right), {\varphi }_{{\mathcal{L}}_{i}}\left(\rho \right)\right)\left({\varrho }_{{\mathcal{L}}_{i}},{\sigma }_{{\mathcal{L}}_{i}}\right)\right\}\in \mathrm{LDF}\left(\wp \right).(i=\{\mathrm{1,2}\} \in \aleph ,$ the GDM satisfied the following properties:

1. $$0\ll {\mathrm{d}}^{g}\left({\mathcal{L}}_{1},{\mathcal{L}}_{2}\right)\ll 1$$
2. $${\mathrm{d}}^{g}\left({\mathcal{L}}_{1},{\mathcal{L}}_{2}\right)=1,$$ iff $${\mathcal{L}}_{1}={\mathcal{L}}_{2}$$.
3. $${\mathrm{d}}^{g}\left({\mathcal{L}}_{1},{\mathcal{L}}_{2}\right)={\mathrm{d}}^{g}\left({\mathcal{L}}_{2},{\mathcal{L}}_{1}\right)$$

#### Entropy measure for LDFs

Suppose $\mathfrak{I}=\{{\mathfrak{I}}_{1},{\mathfrak{I}}_{2},...,{\mathfrak{I}}_{n}\}\in LDF(\rho )$, where ${\mathfrak{I}}_{i}=\left\{\left({\varphi }_{{\mathfrak{I}}_{i}}\left(\rho \right), {\varphi }_{{\mathfrak{I}}_{i}}\left(\rho \right)\right)\left({\varrho }_{{\mathfrak{I}}_{i}},{\sigma }_{{\mathfrak{I}}_{i}}\right)\right\}$ is a LDFs for each $\left(i\in \aleph \right)$. The entropy measure(EM) for LDFs $\mathfrak{I}$ is defined as:

15 $$\mathrm{\rm E}\left(\mathfrak{I}\right)=\frac{1}{n}\sum_{i=1}^{n}\left[\left\{1-d\left(\mathfrak{I},{\mathfrak{I}}^{c}\right)\right\}\frac{1+{\pi }_{\mathfrak{I}}}{2}\right]$$

Suppose $\mathfrak{I}=\{{\mathfrak{I}}_{1},{\mathfrak{I}}_{2},...,{\mathfrak{I}}_{n}\}$ and $\mathcal{K}=\{{\mathcal{K}}_{1},{\mathcal{K}}_{2},...,{\mathcal{K}}_{n}\}\in \mathrm{LDF}(\wp )$ where ${\mathfrak{I}}_{i}=\left\{\left({\varphi }_{{\mathfrak{I}}_{i}}\left(\rho \right), {\varphi }_{{\mathfrak{I}}_{i}}\left(\rho \right)\right)\left({\varrho }_{{\mathfrak{I}}_{i}},{\sigma }_{{\mathfrak{I}}_{i}}\right)\right\}$ and ${\mathcal{K}}_{i}=\left\{\left({\varphi }_{{\mathcal{K}}_{i}}\left(\rho \right), {\varphi }_{{\mathcal{K}}_{i}}\left(\rho \right)\right)\left({\varrho }_{{\mathcal{K}}_{i}},{\sigma }_{{\mathcal{K}}_{i}}\right)\right\}$ $\left(i\in \aleph \right)$. Then, LDFs entropy measure satisfies the following properties:

1. $\mathrm{\rm E}\left(\mathfrak{I}\right)=0$ iff $\mathfrak{I}$ is the crisp set.
2. $$\mathrm{\rm E}\left(\mathfrak{I}\right)\ll \mathrm{\rm E}\left(\mathcal{K}\right)$$ if $$\left(\mathfrak{I}\right)\ll \left(\mathcal{K}\right)$$.
3. $$\mathrm{\rm E}\left(\mathfrak{I}\right)\ll \mathrm{\rm E}\left({\mathfrak{I}}^{c}\right)$$

## Results

In this part, we develop an extended version of the TOPSIS method for the MCDM problem under the LDF information. In this DMM, we have three-part: one calculates the weight of the decision maker using entropy measure; in the second part, we evaluate the weight of criteria and the third part is the decision-making process.

To start the decision-making process, consider $n$ alternatives and $m$ criteria, and the decision-making gives their data information of each alternative based on criteria in the form of LDF numbers. The decision information is given in Table 2

**Table 2:** Linear Diophantine fuzzy decision making.

Alternatives	${G}_{1}$	${G}_{2}$	$\dots$	${G}_{m}$
${A}_{1}$	${\mathcal{l}}_{11}$	${\mathcal{l}}_{12}$	$\dots$	${\mathcal{l}}_{1m}$
${A}_{2}$	${\mathcal{l}}_{21}$	${\mathcal{l}}_{22}$	$\dots$	${\mathcal{l}}_{2m}$
${A}_{3}$	${\mathcal{l}}_{31}$	${\mathcal{l}}_{32}$	$\dots$	${\mathcal{l}}_{3m}$
$\vdots$	$\vdots$	$\vdots$	$\vdots$	$\vdots$
${A}_{n}$	${\mathcal{l}}_{n1}$	${\mathcal{l}}_{n2}$	$\dots$	${\mathcal{l}}_{nm}$

### Find weight of decision experts

**1(a);** In the decision-making process, we use two types of criteria; one is benefited and the other is cost type criteria. We will normalize the decision table by using the following equations

$${\aleph }_{ij}=\left\{\begin{array}{c}{\mathcal{l}}_{ij} if C is benefit criteria\\ {{\mathcal{l}}_{ij}}^{c} if C is Cost criteria,\end{array}\right.$$

where ${{\mathcal{l}}_{ij}}^{c}$ is the complement of LDFV ${\mathcal{l}}_{ij}$.

**2(b);** We will consider LDF weighted aggregation operators to find the group decision ideal solution (GDIS) of each decision expert's information. Using Eq. (16) the information of GIDS is given in Table 3.

**Table 3:** Information of GDIS.

Alternatives	${G}_{1}$	${G}_{2}$	$\dots$	${G}_{m}$
${A}_{1}$	${GI}_{11}$	${GI}_{12}$	$\dots$	${GI}_{1m}$
${A}_{2}$	${GI}_{21}$	${GI}_{22}$	$\dots$	${GI}_{2m}$
${A}_{3}$	${GI}_{31}$	${GI}_{32}$	$\dots$	${GI}_{3m}$
$\vdots$	$\vdots$	$\vdots$	$\vdots$	$\vdots$
${A}_{n}$	${GI}_{n1}$	${GI}_{n2}$	$\dots$	${GI}_{nm}$

16 $${\mathrm{GDIS }}_{ij}=\left\{\begin{array}{c}\left(1-{\left(\sum_{i=1}^{n}{\omega }_{i}\left({\left(1-{\varphi }_{{\mathcal{L}}_{i}}\right)}^{-\lambda }-1\right)+1\right)}^{-\frac{1}{\lambda }},{\left(\sum_{i=1}^{n}{\omega }_{i}\left({\left({ \psi }_{{\mathcal{L}}_{i}}\right)}^{-\lambda }-1\right)+1\right)}^{-\frac{1}{\lambda }}\right),\\ \left(1-{\left(\sum_{i=1}^{n}{\omega }_{i}\left({\left(1-{\varrho }_{i}\right)}^{-\lambda }-1\right)+1\right)}^{-\frac{1}{\lambda }}, {\left(\sum_{i=1}^{n}{\omega }_{i}\left({\left({\sigma }_{i}\right)}^{-\lambda }-1\right)+1\right)}^{-\frac{1}{\lambda }}\right)\end{array}\right\}$$

**1(c)** Calculate the group right ideal solution (GRIS) and the group left ideal solution (GLIS) as given in Tables 4 and 5.

**Table 4:** Information of GRIS.

Alternatives	${G}_{1}$	${G}_{2}$	$\dots$	${G}_{m}$
${A}_{1}$	${RIS}_{11}$	${RIS}_{12}$	$\dots$	${RIS}_{1m}$
${A}_{2}$	${RIS}_{21}$	${RIS}_{22}$	$\dots$	${RIS}_{2m}$
${A}_{3}$	${RIS}_{31}$	${RIS}_{32}$	$\dots$	${RIS}_{3m}$
$\vdots$	$\vdots$	$\vdots$	$\vdots$	$\vdots$
${A}_{n}$	${RIS}_{n1}$	${RIS}_{n2}$	$\dots$	${RIS}_{nm}$

Where

17 $${RIS}_{ij}=\left\{{\aleph }_{ij}: {}_{k }{}^{max}\left[SC{({\aleph }_{ij})}^{k}\right]\right\}$$

**Table 5:** Information of GLIS.

Alternatives	${G}_{1}$	${G}_{2}$	$\dots$	${G}_{m}$
${A}_{1}$	${LIS}_{11}$	${LIS}_{12}$	$\dots$	${LIS}_{1m}$
${A}_{2}$	${LIS}_{21}$	${LIS}_{22}$	$\dots$	${LIS}_{2m}$
${A}_{3}$	${LIS}_{31}$	${LIS}_{32}$	$\dots$	${LIS}_{3m}$
$\vdots$	$\vdots$	$\vdots$	$\vdots$	$\vdots$
${A}_{n}$	${LIS}_{n1}$	${LIS}_{n2}$	$\dots$	${LIS}_{nm}$

Where

18 $${LIS}_{ij}=\left\{{\aleph }_{ij}: {}_{i }{}^{min}\left[SC{({\aleph }_{ij})}^{k}\right]\right\}$$

**1(d):** To evaluate the distance measure between the normalized decision matrix and GDIS, GRIS, and GLIS by using the distance measure equation. then further, the closeness indices are evaluated by the following equations;

19 $$\mathrm{DGRIS}={\left(\frac{1}{4n}\sum_{i=1}^{n}\left({\left|{\varphi }_{{\mathcal{l}}_{ij}}\left({\rho }_{i}\right)-{\varphi }_{{RIS}_{ij}}\left({\rho }_{i}\right)\right|}^{\Upsilon }+{\left|{ \psi }_{{\varphi }_{{\mathcal{l}}_{ij}}}\left({\rho }_{i}\right)-{ \psi }_{{RIS}_{ij}}\left({\rho }_{i}\right)\right|}^{\Upsilon }+{\left|{\varrho }_{{\mathcal{l}}_{ij}}-{\varrho }_{{RIS}_{ij}}\right|}^{\Upsilon }+{\left|{\sigma }_{{\mathcal{l}}_{ij}}-{\sigma }_{{RIS}_{ij}}\right|}^{\Upsilon }\right)\right)}^{\frac{1}{\Upsilon }}$$

20 $$\mathrm{DG}L\mathrm{IS}={\left(\frac{1}{4n}\sum_{i=1}^{n}\left({\left|{\varphi }_{{\mathcal{l}}_{ij}}\left({\rho }_{i}\right)-{\varphi }_{{LIS}_{ij}}\left({\rho }_{i}\right)\right|}^{\Upsilon }+{\left|{ \psi }_{{\varphi }_{{\mathcal{l}}_{ij}}}\left({\rho }_{i}\right)-{ \psi }_{{LIS}_{ij}}\left({\rho }_{i}\right)\right|}^{\Upsilon }+{\left|{\varrho }_{{\mathcal{l}}_{ij}}-{\varrho }_{{LIS}_{ij}}\right|}^{\Upsilon }+{\left|{\sigma }_{{\mathcal{l}}_{ij}}-{\sigma }_{{LIS}_{ij}}\right|}^{\Upsilon }\right)\right)}^{\frac{1}{\Upsilon }}$$

21 $$\mathrm{DGDIS}={\left(\frac{1}{4n}\sum_{i=1}^{n}\left({\left|{\varphi }_{{\mathcal{l}}_{ij}}\left(\rho \right)-{\varphi }_{{GI}_{ij}}\left(\rho \right)\right|}^{\Upsilon }+{\left|{ \psi }_{{\mathcal{l}}_{ij}}\left({\rho }_{i}\right)-{ \psi }_{{GI}_{ij}}\left({\rho }_{i}\right)\right|}^{\Upsilon }+{\left|{\varrho }_{{\mathcal{l}}_{ij}}-{\varrho }_{{GI}_{ij}}\right|}^{\Upsilon }+{\left|{\sigma }_{{\mathcal{l}}_{ij}}-{\sigma }_{{GI}_{ij}}\right|}^{\Upsilon }\right)\right)}^{\frac{1}{\Upsilon }}$$

**1(e)** The closeness indices (CIs) are computed as follows:

22 $$C{I}^{k}= \frac{\sum_{i=1}^{n}\mathrm{DGRIS}+\sum_{i=1}^{n}\mathrm{DGLIS}}{\sum_{i=1}^{n}\mathrm{DGDIS}+\sum_{i=1}^{n}\mathrm{DGRIS}+\sum_{i=1}^{n}\mathrm{DGLIS}}$$

**1(f)** Find the weight of the decision maker by using the following equation;

23 $${w}_{k}= \frac{C{I}^{k}}{\sum_{i=1}^{l}C{I}^{k}}, k=\mathrm{1,2},\dots ,l$$

### Find weight of criteria

**2(a):** First we find the revised aggregated normalized group decision information by using LDFWA operators with weights ${w}_{k},k=\mathrm{1,2},\dots ,l$. Then we find the LD-entropy measure of each criterion as follows.

24 $${RIS}_{ij}=\left\{\begin{array}{c}\left(1-{\left(\sum_{i=1}^{n}{w}_{k}\left({\left(1-{\varphi }_{{\mathcal{L}}_{i}}\right)}^{-\lambda }-1\right)+1\right)}^{-\frac{1}{\lambda }},{\left(\sum_{i=1}^{n}{w}_{k}\left({\left({ \psi }_{{\mathcal{L}}_{i}}\right)}^{-\lambda }-1\right)+1\right)}^{-\frac{1}{\lambda }}\right),\\ \left(1-{\left(\sum_{i=1}^{n}{w}_{k}\left({\left(1-{\varrho }_{i}\right)}^{-\lambda }-1\right)+1\right)}^{-\frac{1}{\lambda }}, {\left(\sum_{i=1}^{n}{w}_{k}\left({\left({\sigma }_{i}\right)}^{-\lambda }-1\right)+1\right)}^{-\frac{1}{\lambda }}\right)\end{array}\right\}$$

Using the following Eq. (25) LDF entropy measure corresponding to each Criteria is computed as follows:

25 $$E\left({C}_{j}\right)={\sum }_{i=1}^{n}{E}_{ij}\left\{\mathrm{RGRIS}\right\}.$$

**2(b)** Find the weight of the criteria ${\gamma }_{j}$ by using the following equation;

26 $${\gamma }_{j}=\frac{1-E\left({C}_{j}\right)}{n-\sum_{j=1}^{m}E\left({C}_{j}\right)},j=\mathrm{1,2},\dots ,m$$

### Decision-making process

**3(a)** Find the Collective Normalized decision information of all experts by using the LDFWA operator $w=\left({w}_{1},{w}_{2},\dots ,{w}_{e}\right)$. In Table 6, the aggregated normalized information of LDF decision has discussed.

**Table 6:** Aggregated Normalized LDF Decision information.

Alternatives	${G}_{1}$	${G}_{2}$	$\dots$	${G}_{m}$
${A}_{1}$	${\eta }_{11}$	${\eta }_{12}$	$\dots$	${\eta }_{1m}$
${A}_{2}$	${\eta }_{21}$	${\eta }_{22}$	$\dots$	${\eta }_{2m}$
${A}_{3}$	${\eta }_{31}$	${\eta }_{32}$	$\dots$	${\eta }_{3m}$
$\vdots$	$\vdots$	$\vdots$	$\vdots$	$\vdots$
${A}_{n}$	${\eta }_{n1}$	${\eta }_{n2}$	$\dots$	${\eta }_{nm}$

27 $${NDM}_{ij}=\left\{\begin{array}{c}\left(1-{\left({\gamma }_{j}\left({\left(1-{\varphi }_{{\mathcal{L}}_{i}}\right)}^{-\lambda }-1\right)+1\right)}^{-\frac{1}{\lambda }},{\left({\gamma }_{j}\left({\left({ \psi }_{{\mathcal{L}}_{i}}\right)}^{-\lambda }-1\right)+1\right)}^{-\frac{1}{\lambda }}\right),\\ \left(1-{\left({\gamma }_{j}\left({\left(1-{\varrho }_{i}\right)}^{-\lambda }-1\right)+1\right)}^{-\frac{1}{\lambda }}, {\left({\gamma }_{j}\left({\left({\sigma }_{i}\right)}^{-\lambda }-1\right)+1\right)}^{-\frac{1}{\lambda }}\right)\end{array}\right\}$$

**3 (b)** Find the PIS and NIS by using the following equations;

28 $$PIS=\left\{{\eta }_{ij}: {}_{i }{}^{max}SC\left({\eta }_{ij}\right)\right\} and NIS=\left\{{\eta }_{ij}: {}_{i }{}^{min}SC\left({\eta }_{ij}\right)\right\}$$

**3(c)** Find the distance measure between $PIS$ and each alternative (DIS^+^) by using the weight distance measure of two LDFSs and also find the distance measure between $NIS$ and each alternative (DIS^−^) by using weight distance equation;

29 $${\mathrm{DIS}}_{\mathrm{i}}^{+(\mathrm{K})}={\left(\frac{1}{4\mathrm{n}}\sum_{\mathrm{i}=1}^{\mathrm{n}}{\upgamma }_{\mathrm{j}}\left({\left|{\mathrm{\varphi }}_{{\mathrm{NDM}}_{\mathrm{ij}}}\left({\uprho }_{\mathrm{i}}\right)-{\mathrm{\varphi }}_{{\mathrm{PIS}}_{\mathrm{j}}}\left({\uprho }_{\mathrm{i}}\right)\right|}^{\Upsilon }+{\left|{\uppsi }_{{\mathrm{NDM}}_{\mathrm{ij}}}\left({\uprho }_{\mathrm{i}}\right)-{\uppsi }_{{\mathrm{PIS}}_{\mathrm{j}}}\left(\uprho \right)\right|}^{\Upsilon }+{\left|{\mathrm{\varrho }}_{{\mathrm{NDM}}_{\mathrm{ij}}}-{\mathrm{\varsigma }}_{{\mathrm{PIS}}_{\mathrm{j}}}\right|}^{\Upsilon }+{\left|{\mathrm{\varsigma }}_{{\mathrm{NDM}}_{\mathrm{ij}}}-{\uptau }_{{\mathrm{PIS}}_{\mathrm{j}}}\right|}^{\Upsilon }\right)\right)}^{\frac{1}{\Upsilon }}$$

30 $${\mathrm{DIS}}_{\mathrm{i}}^{-(\mathrm{K})}={\left(\frac{1}{4\mathrm{n}}\sum_{\mathrm{i}=1}^{\mathrm{n}}{\upgamma }_{\mathrm{j}}\left({\left|{\mathrm{\varphi }}_{{\mathrm{NDM}}_{\mathrm{ij}}}\left({\uprho }_{\mathrm{i}}\right)-{\mathrm{\varphi }}_{{\mathrm{NIS}}_{\mathrm{j}}}\left({\uprho }_{\mathrm{i}}\right)\right|}^{\Upsilon }+{\left|{\uppsi }_{{\mathrm{NDM}}_{\mathrm{ij}}}\left({\uprho }_{\mathrm{i}}\right)-{\uppsi }_{{\mathrm{NIS}}_{\mathrm{j}}}\left(\uprho \right)\right|}^{\Upsilon }+{\left|{\mathrm{\varrho }}_{{\mathrm{NDM}}_{\mathrm{ij}}}-{\mathrm{\varsigma }}_{{\mathrm{NIS}}_{\mathrm{j}}}\right|}^{\Upsilon }+{\left|{\mathrm{\varsigma }}_{{\mathrm{NDM}}_{\mathrm{ij}}}-{\uptau }_{{\mathrm{NIS}}_{\mathrm{j}}}\right|}^{\Upsilon }\right)\right)}^{\frac{1}{\Upsilon }}$$

**3(d)** Find the closeness indices by the following equation

31 $$C\left({A}_{i}\right)=\frac{{DIS}^{-}}{{DIS}^{+}{DIS}^{-}}$$

**3(e)** Find the ranking of the optimal alternative by descending order of closeness indices, the optimal alternative will be maximum of closeness indices.

Final closeness indices:

32 $$FC\left({A}_{i}\right)=\sum_{k=1}^{e}{w}_{k}C\left({A}_{i}^{k}\right)$$

**Figure 1:**
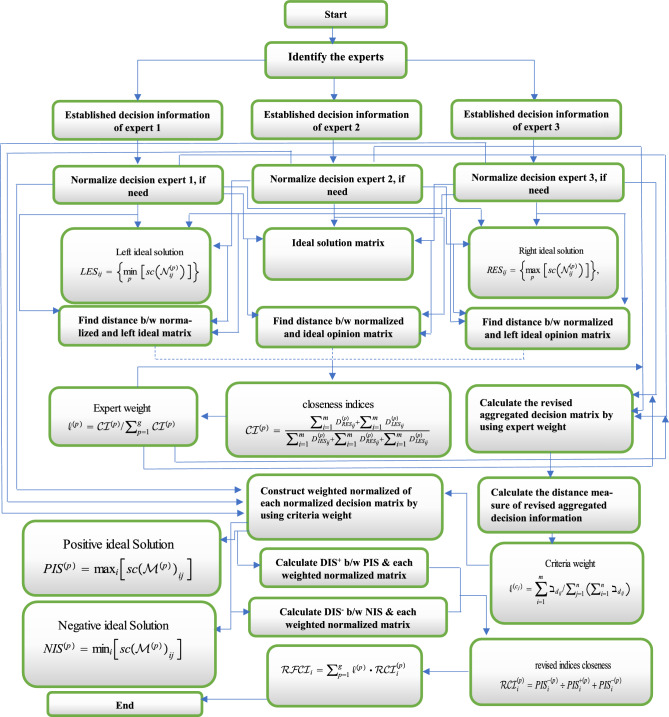
The flow chart of decision support systems.

The flow chart of the proposed Exended-TOPSIS method for decision-making problems under the LDFNs is given in Fig. 1.

### Application to optimal classifier selection in EEG

Nowadays, a human mental disorder or depression is a major ill in the world. Psychological illness has various characteristics, one of which is an important and consistent low mood. Mental disorders or depression are increasing due to a highly busy life. Depression is a disorder that is affecting more and more people and Persistent depression, depression and frustration are major medical manifestations, in which suicidal tendencies appear in severe cases. At the end of 2020, the World Health Organization (WHO) reported that 350 million people were affected by depression and that it was the second largest disease in the world after heart disease^1^. In the middle of the 19th Century, depression properly has studied as a mental disorder disease. Beck, in the 1960s, the theory of cognitive discovered the breakdown in depression^2^ and according to this theory, depressed people have negative thoughts about themselves and the people around them. However, because the etiology of depression is unknown and the manifestations are difficult, clinical diagnosis has been hampered. Physicians use to diagnose this disease by using different ways of diagnostic and ordinary tests, which these different tests give different results of diagnostic. This concludes that depressed patients with outward signs of trust in self-care to implement a management plan of treatment, and there is no such physical signal to apply as a measurable constraint. This is very hard to succeed in an initial checkup, diagnosis, and anticipation of depression. With the rapid growth of technology of cognitive science and sensor technology in recent years, researchers can apply electroencephalograms (EEG) to record brain activities as physiological signals. The clinical record of disorderliness is measured by Electroephalogrphy (EEG). Depression and brain movement have a significant connection, which may be measured and recorded using EEG physiological signals. EEG recordings can be used in a variety of clinical brain function tests.

EEG signals are used to examine brain activity, according to a large quantity of research^4–7^. Recently, the sample entropy (SampEn), bispectrum entropy (Ph), approximate entropy (ApEn), and renyi entropy have been studied for classifier selection of EEG signals for depression and classified into different classes of depression EEG signals. To facilitate monitoring of diagnosis and treatment, more information is needed about the procedures that take place in the brain before and during stress. The proposed intelligent decision technique analyzes the k-nearest neighbor algorithm (k-NN), Gaussian mixture models (GMM), Decision tree (DT), Naïve Bayes classification (NBC), and Probabilistic neural network (PNN) of EEG signals of depression using the SampEn, Ph, ApEn, and REN under the linear Diophantine fuzzy information.

The decision experts finalized the following method for classifier selection P1: k-nearest neighbor algorithm (k-NN), P2: Gaussian mixture models (GMM), P3: Decision tree (DT), P4: Naïve Bayes classification (NBC) and P5: Probabilistic neural network (PNN). The classifier is further evaluated by decision experts based on criteria C1: Sample Entropy (SampEn), C2: Spectral Entropy C3: Bispectrum Entropy (Ph), C4: Approximate Entropy (ApEn), and C5: Renyi Entropy. The experts the five classifiers of EEG based on five given criteria and the detailed information are given in Tables 7, 8, 9.

**Step-1** (a):

**Table 7:** The expert Information Matrix in the form of linear Diophantine fuzzy values:

	C1	C2	C3	C4	C5
P1	(0.8,0.9)	(0.8,0.9)	(0.1,0.1)	(0.9,0.1)	(0.9,0.1)
P2	(0.3,0.9)	(0.3,0.2)	(0.9,0.5)	(0.7,0.9)	(0.9,0.1)
P3	(0.2,0.1)	(0.2,0.9)	(0.3,0.7)	(0.4,0.2)	(0.2,0.9)
P4	(0.7,0.6)	(0.8,0.3)	(0.5,0.8)	(0.4,0.4)	(0.8,0.9)
P5	(0.6,0.7)	(0.1,0.3)	(0.6,0.1)	(0.8,0.7)	(0.9,0.1)

**Table 8:** The expert Information Matrix in the form of linear Diophantine fuzzy values.

	C1	C2	C3	C4	C5
P1	(0.7,0.7)	(0.9,0.1)	(0.9,0.8)	(0.9,0.1)	(0.8,0.9)
P2	(0.5,0.6)	(0.2,0.9)	(0.3,0.1)	(0.2,0.9)	(0.4,0.9)
P3	(0.2,0.9)	(0.5,0.3)	(0.5,0.2)	(0.3,0.3)	(0.5,0.6)
P4	(0.1,0.5)	(0.8,0.9)	(0.9,0.8)	(0.9,0.9)	(0.7,0.8)
P5	(0.3,0.6)	(0.9,1)	(0.9,0.8)	(0.6,0.9)	(0.2,0.9)

**Table 9:** The expert Information Matrix in the form of linear Diophantine fuzzy values.

	C1	C2	C3	C4	C5
P1	(0.2,0.8)	(0.3,0.8)	(0.2,0.5)	(0.8,0.8)	(0.7,0.9)
P2	(0.7,0.8)	(0.4,0.9)	(0.1,0.5)	(0.9,0.9)	(0.2,0.1)
P3	(0.3,0.6)	(0.6,0.4)	(0.3,0.6)	(0.4,0.9)	(0.8,0.3)
P4	(0.2,0.9)	(0.7,0.8)	(0.8,0.9)	(0.9,0.4)	(0.7,0.6)
P5	(0.5,0.4)	(0.2,0.9)	(0.8,0.9)	(0.9,0.4)	(0.6,0.7)

**Step-1(b).** As we know that all criteria are beneficial so there is no need for normalization:

**Step -2(a):** Using Eq. (16) $LDFWCA$ operator, GDIS is calculated in Table 10.

**Table 10:** The GDIS.

	${C}_{1}$	${C}_{2}$	${C}_{3}$	${C}_{4}$	${C}_{5}$
${P}_{1}$	$$\left(\begin{array}{c}0.684, 0.302\\ \mathrm{0.834,0.302}\end{array}\right)$$	$$\left(\begin{array}{c}0.815, 0.302\\ \mathrm{0.812,0.302}\end{array}\right)$$	$$\left(\begin{array}{c}0.755, 0.302\\ \mathrm{0.627,0.302}\end{array}\right)$$	$$\left(\begin{array}{c}0.878, 0.302\\ \mathrm{0.582,0.302}\end{array}\right)$$	$$\left(\begin{array}{c}0.834, 0.302\\ \mathrm{0.856,0.302}\end{array}\right)$$
${P}_{2}$	$$\left(\begin{array}{c}0.553, 0.302\\ \mathrm{0.827,0.302}\end{array}\right)$$	$$\left(\begin{array}{c}0.307, 0.302\\ \mathrm{0.857,0.302}\end{array}\right)$$	$$\left(\begin{array}{c}0.758, 0.302\\ \mathrm{0.410,0.302}\end{array}\right)$$	$$\left(\begin{array}{c}0.792, 0.302\\ \mathrm{0.899,0.302}\end{array}\right)$$	$$\left(\begin{array}{c}0.765, 0.302\\ \mathrm{0.752,0.302}\end{array}\right)$$
${P}_{3}$	$$\left(\begin{array}{c}0.234, 0.302\\ \mathrm{0.777,0.302}\end{array}\right)$$	$$\left(\begin{array}{c}0.475, 0.302\\ \mathrm{0.769,0.302}\end{array}\right)$$	$$\left(\begin{array}{c}0.379, 0.302\\ \mathrm{0.574,0.302}\end{array}\right)$$	$$\left(\begin{array}{c}0.367, 0.302\\ \mathrm{0.761,0.302}\end{array}\right)$$	$$\left(\begin{array}{c}0.634, 0.302\\ \mathrm{0.782,0.302}\end{array}\right)$$
${P}_{4}$	$$\left(\begin{array}{c}0.470, 0.302\\ \mathrm{0.791,0.302}\end{array}\right)$$	$$\left(\begin{array}{c}0.773, 0.302\\ \mathrm{0.815,0.302}\end{array}\right)$$	$$\left(\begin{array}{c}0.822, 0.302\\ \mathrm{0.848,0.302}\end{array}\right)$$	$$\left(\begin{array}{c}0.860, 0.302\\ \mathrm{0.773,0.302}\end{array}\right)$$	$$\left(\begin{array}{c}0.740, 0.302\\ \mathrm{0.827,0.302}\end{array}\right)$$
${P}_{5}$	$$\left(\begin{array}{c}0.491, 0.302\\ \mathrm{0.597,0.302}\end{array}\right)$$	$$\left(\begin{array}{c}0.755, 0.302\\ \mathrm{0.758,0.302}\end{array}\right)$$	$$\left(\begin{array}{c}0.827, 0.302\\ \mathrm{0.812,0.302}\end{array}\right)$$	$$\left(\begin{array}{c}0.827, 0.302\\ \mathrm{0.798,0.302}\end{array}\right)$$	$$\left(\begin{array}{c}0.780, 0.302\\ \mathrm{0.790,0.302}\end{array}\right)$$

**Step -2(b):** The Group Right Ideal Solution and Group Lift Ideal Solution are calculated in Table 11.

**Table 11:** GRIS and GLIS.

	${{\varvec{C}}}_{1}$	${{\varvec{C}}}_{2}$	${{\varvec{C}}}_{3}$	${{\varvec{C}}}_{4}$	${{\varvec{C}}}_{5}$
GRIS					
${{\varvec{P}}}_{1}$	$\left(0.7, 0.7\right)$	$\left(0.7, 0.7\right)$	$\left(0.9, 0.8\right)$	$\left(0.9, 0.1\right)$	$\left(0.9, 0.1\right)$
${{\varvec{P}}}_{2}$	$\left(0.3, 0.9\right)$	$\left(0.3, 0.2\right)$	$\left(0.9, 0.5\right)$	$\left(0.9, 0.9\right)$	$\left(0.9, 0.1\right)$
${{\varvec{P}}}_{3}$	$\left(0.2, 0.1\right)$	$\left(0.5, 0.3\right)$	$\left(0.5, 0.2\right)$	$\left(0.4, 0.2\right)$	$\left(0.8, 0.3\right)$
${{\varvec{P}}}_{4}$	$\left(0.7, 0.6\right)$	$\left(0.8, 0.3\right)$	$\left(0.9, 0.8\right)$	$\left(0.9, 0.4\right)$	$\left(0.7, 0.6\right)$
${{\varvec{P}}}_{5}$	$\left(0.6, 0.7\right)$	$\left(0.9, 0.1\right)$	$\left(0.6, 0.1\right)$	$\left(0.9, 0.4\right)$	$\left(0.9, 0.1\right)$
GLIS					
${{\varvec{P}}}_{1}$	$\left(0.2, 0.8\right)$	$\left(0.3, 0.8\right)$	$\left(0.2, 0.5\right)$	$\left(0.8, 0.8\right)$	$\left(0.9, 0.1\right)$
${{\varvec{P}}}_{2}$	$\left(0.5, 0.6\right)$	$\left(0.2, 0.9\right)$	$\left(0.1, 0.5\right)$	$\left(0.2, 0.9\right)$	$\left(0.9, 0.1\right)$
${{\varvec{P}}}_{3}$	$\left(0.2, 0.9\right)$	$\left(0.2, 0.9\right)$	$\left(0.3, 0.7\right)$	$\left(0.4, 0.9\right)$	$\left(0.8, 0.3\right)$
${{\varvec{P}}}_{4}$	$\left(0.2, 0.9\right)$	$\left(0.8, 0.9\right)$	$\left(0.5, 0.8\right)$	$\left(0.4, 0.4\right)$	$\left(0.7, 0.6\right)$
${{\varvec{P}}}_{5}$	$\left(0.3, 0.6\right)$	$\left(0.2, 0.9\right)$	$\left(0.8, 0.9\right)$	$\left(0.6, 0.9\right)$	$\left(0.9, 0.1\right)$

**Step-2(c).** $\mathrm{DGDIS}$, $\mathrm{DGRIS}$**,** and $\mathrm{DG}L\mathrm{IS}$ are computed by using Eqs. (19), (20) & (21) as follows in Table 12.

**Table 12:** Distance measure of all alternative.

	${P}_{1}$	${P}_{2}$	${P}_{3}$	${P}_{4}$	${P}_{5}$
$$\mathrm{DGDIS}$$					
${\mathrm{DM}}_{1}$	0.247	0.221	0.233	0.202	0.543
${\mathrm{DM}}_{2}$	0.202	0.209	0.181	0.113	0.213
${\mathrm{DM}}_{3}$	0.212	0.248	0.154	0.119	0.170
${\mathrm{DM}}_{1}$	0.247	0.044	0.270	0.159	0.197
$$\mathrm{DGRIS}$$					
${\mathrm{DM}}_{1}$	0.247	0.044	0.270	0.159	0.197
${\mathrm{DM}}_{2}$	0.229	0.355	0.204	0.225	0.327
${\mathrm{DM}}_{3}$	0.330	0.300	0.220	0.176	0.343
$$\mathrm{DGLIS}$$					
${\mathrm{DM}}_{1}$	0.314	0.347	0.237	0.189	0.343
${\mathrm{DM}}_{2}$	0.331	0.010	0.253	0.203	0.239
${\mathrm{DM}}_{3}$	0.000	0.253	0.248	0.143	0.176

**Step -2 (d,f):** The closeness indices (CIs) and weight of the decision maker are calculated as follows in Table 13.

**Table 13:** The closeness indices (CIs) and weight of decision maker.

(CIs)					
$\mathbf{C}{\mathbf{I}}_{1}$					*0.613*
$\mathbf{C}{\mathbf{I}}_{2}$					0.728
$\mathbf{C}{\mathbf{I}}_{3}$					0.707
$${{\varvec{w}}}_{{\varvec{k}}}$$					
${{\varvec{w}}}_{1}$					0.2995
${{\varvec{w}}}_{2}$					0.3553
${{\varvec{w}}}_{3}$					0.3452

**Step-3(a):** The revised group decision (RGDIS) matrix is computed as follows in Table 14.

**Table 14:** Revised GDIS.

RGDIS	${{\varvec{C}}}_{1}$	${{\varvec{C}}}_{2}$	${{\varvec{C}}}_{3}$	${{\varvec{C}}}_{4}$	${{\varvec{C}}}_{5}$
${{\varvec{P}}}_{1}$	$\left(0.678, 0.830\right)$	$\left(0.819, 0.804\right)$	$\left(0.768, 0.642\right)$	$\left(0.879, 0.592\right)$	$\left(\mathrm{0.831,0.863}\right)$
${{\varvec{P}}}_{2}$	$\left(0.563, 0.821\right)$	$\left(0.309, 0.864\right)$	$\left(0.742, 0.406\right)$	$\left(0.795, 0.900\right)$	$\left(\mathrm{0.751,0.765}\right)$
${{\varvec{P}}}_{3}$	$\left(0.237, 0.789\right)$	$\left(0.486, 0.754\right)$	$\left(0.387, 0.566\right)$	$\left(0.367, 0.769\right)$	$\left(0.644, 0.771\right)$
${{\varvec{P}}}_{4}$	$\left(0.451, 0.796\right)$	$\left(0.773, 0.824\right)$	$\left(0.829, 0.851\right)$	$\left(0.866, 0.783\right)$	$\left(\mathrm{0.739,0.822}\right)$
${{\varvec{P}}}_{5}$	$\left(0.486, 0.593\right)$	$\left(0.768, 0.766\right)$	$\left(0.834, 0.820\right)$	$\left(0.828, 0.804\right)$	$\left(\mathrm{0.767,0.801}\right)$

**Step-3 (**b,c**):** Usig Eq. (25) LDF entropy measure corresponding to each criterion, and the weight of criteria is computed as follows in Table 15.

**Table 15:** Entropy measure and weight of criteria.

$${\varvec{E}}\left({{\varvec{C}}}_{{\varvec{j}}}\right)$$			
${\varvec{E}}\left({{\varvec{C}}}_{1}\right)$	0.15964	${\gamma }_{1}$	0.2046
${\varvec{E}}\left({{\varvec{C}}}_{2}\right)$	0.16472	${\gamma }_{2}$	0.2036
${\varvec{E}}\left({{\varvec{C}}}_{3}\right)$	0.18522	${\gamma }_{3}$	0.1970
${\varvec{E}}\left({{\varvec{C}}}_{4}\right)$	0.17195	${\gamma }_{4}$	0.2017
${\varvec{E}}\left({{\varvec{C}}}_{5}\right)$	0.20099	${\gamma }_{5}$	0.1931

**Step-4(a)**: The weighted normalized decision matrix is calculated as follows in Table 16.

**Table 16:** Weight-normalized decision ${DM}_{1}$, ${DM}_{2}$, ${DM}_{3}$ information $(DM{(N}_{ij}^{1}$)),$(DM{(N}_{ij}^{2}$)), and $(DM{(N}_{ij}^{3}$)).

${\varvec{D}}{{\varvec{M}}}_{1}$	${{\varvec{C}}}_{1}$	${{\varvec{C}}}_{2}$	${{\varvec{C}}}_{3}$	${{\varvec{C}}}_{4}$	${{\varvec{C}}}_{5}$
${{\varvec{P}}}_{1}$	$$\left(\begin{array}{c}0.450, 0.676\\ \mathrm{0.648,0.676}\end{array}\right)$$	$$\left(\begin{array}{c}0.448, 0.677\\ \mathrm{0.646,0.677}\end{array}\right)$$	$$\left(\begin{array}{c}0.021, 0.685\\ \mathrm{0.021,0.685}\end{array}\right)$$	$$\left(\begin{array}{c}0.644, 0.679\\ \mathrm{0.021,0.679}\end{array}\right)$$	$$\left(\begin{array}{c}0.634, 0.689\\ \mathrm{0.021,0.689}\end{array}\right)$$
${{\varvec{P}}}_{2}$	$$\left(\begin{array}{c}0.080, 0.676\\ \mathrm{0.648,0.676}\end{array}\right)$$	$$\left(\begin{array}{c}0.080, 0.677\\ \mathrm{0.048,0.677}\end{array}\right)$$	$$\left(\begin{array}{c}0.639, 0.685\\ \mathrm{0.164,0.685}\end{array}\right)$$	$$\left(\begin{array}{c}0.320, 0.679\\ \mathrm{0.644,0.679}\end{array}\right)$$	$$\left(\begin{array}{c}0.634, 0.689\\ \mathrm{0.021,0.689}\end{array}\right)$$
${{\varvec{P}}}_{3}$	$$\left(\begin{array}{c}0.048, 0.676\\ \mathrm{0.022,0.676}\end{array}\right)$$	$$\left(\begin{array}{c}0.048, 0.677\\ \mathrm{0.646,0.677}\end{array}\right)$$	$$\left(\begin{array}{c}0.077, 0.685\\ \mathrm{0.314,0.685}\end{array}\right)$$	$$\left(\begin{array}{c}0.118, 0.679\\ \mathrm{0.048,0.679}\end{array}\right)$$	$$\left(\begin{array}{c}0.046, 0.689\\ \mathrm{0.634,0.689}\end{array}\right)$$
${{\varvec{P}}}_{4}$	$$\left(\begin{array}{c}0.323, 0.676\\ \mathrm{0.234,0.676}\end{array}\right)$$	$$\left(\begin{array}{c}0.448, 0.677\\ \mathrm{0.080,0.677}\end{array}\right)$$	$$\left(\begin{array}{c}0.164, 0.685\\ 0.44\mathrm{0,0.685}\end{array}\right)$$	$$\left(\begin{array}{c}0.118, 0.679\\ \mathrm{0.118,0.679}\end{array}\right)$$	$$\left(\begin{array}{c}0.435, 0.689\\ \mathrm{0.634,0.689}\end{array}\right)$$
${{\varvec{P}}}_{5}$	$$\left(\begin{array}{c}0.234, 0.676\\ \mathrm{0.323,0.676}\end{array}\right)$$	$$\left(\begin{array}{c}0.022, 0.677\\ \mathrm{0.080,0.677}\end{array}\right)$$	$$\left(\begin{array}{c}0.228, 0.685\\ \mathrm{0.021,0.685}\end{array}\right)$$	$$\left(\begin{array}{c}0.446, 0.679\\ \mathrm{0.320,0.679}\end{array}\right)$$	$$\left(\begin{array}{c}0.634, 0.689\\ \mathrm{0.021,0.689}\end{array}\right)$$
${\varvec{D}}{{\varvec{M}}}_{2}$	${C}_{1}$	${C}_{2}$	${C}_{3}$	${C}_{4}$	${C}_{5}$
${{\varvec{P}}}_{1}$	$$\left(\begin{array}{c}0.323, 0.676\\ 0.323, 0.676\end{array}\right)$$	$$\left(\begin{array}{c}0.646, 0.677\\ 0.022, 0.677\end{array}\right)$$	$$\left(\begin{array}{c}0.639, 0.685\\ \mathrm{0.648,0.685}\end{array}\right)$$	$$\left(\begin{array}{c}0.644, 0.679\\ \mathrm{0.021,0.679}\end{array}\right)$$	$$\left(\begin{array}{c}0.435, 0.689\\ \mathrm{0.634,0.689}\end{array}\right)$$
${{\varvec{P}}}_{2}$	$$\left(\begin{array}{c}0.169, 0.676\\ \mathrm{0.234,0.676}\end{array}\right)$$	$$\left(\begin{array}{c}0.048, 0.677\\ \mathrm{0.646,0.677}\end{array}\right)$$	$$\left(\begin{array}{c}0.077, 0.685\\ \mathrm{0.021,0.685}\end{array}\right)$$	$$\left(\begin{array}{c}0.048, 0.679\\ \mathrm{0.644,0.679}\end{array}\right)$$	$$\left(\begin{array}{c}0.114, 0.689\\ \mathrm{0.634,0.689}\end{array}\right)$$
${{\varvec{P}}}_{3}$	$$\left(\begin{array}{c}0.048, 0.676\\ \mathrm{0.648,0.676}\end{array}\right)$$	$$\left(\begin{array}{c}0.169, 0.677\\ \mathrm{0.080,0.677}\end{array}\right)$$	$$\left(\begin{array}{c}0.164, 0.685\\ \mathrm{0.046,0.685}\end{array}\right)$$	$$\left(\begin{array}{c}0.079, 0.679\\ \mathrm{0.079,0.679}\end{array}\right)$$	$$\left(\begin{array}{c}0.161, 0.689\\ \mathrm{0.224,0.689}\end{array}\right)$$
${{\varvec{P}}}_{4}$	$$\left(\begin{array}{c}0.022, 0.676\\ \mathrm{0.169,0.676}\end{array}\right)$$	$$\left(\begin{array}{c}0.448, 0.677\\ \mathrm{0.646,0.677}\end{array}\right)$$	$$\left(\begin{array}{c}0.639, 0.685\\ \mathrm{0.440,0.685}\end{array}\right)$$	$$\left(\begin{array}{c}0.664, 0.679\\ \mathrm{0.664,0.679}\end{array}\right)$$	$$\left(\begin{array}{c}0.310, 0.689\\ \mathrm{0.435,0.689}\end{array}\right)$$
${{\varvec{P}}}_{5}$	$$\left(\begin{array}{c}0.080, 0.676\\ \mathrm{0.234,0.676}\end{array}\right)$$	$$\left(\begin{array}{c}0.646, 0.677\\ \mathrm{0.022,0.677}\end{array}\right)$$	$$\left(\begin{array}{c}0.639, 0.685\\ \mathrm{0.440,0.685}\end{array}\right)$$	$$\left(\begin{array}{c}0.232, 0.679\\ \mathrm{0.644,0.679}\end{array}\right)$$	$$\left(\begin{array}{c}0.046, 0.689\\ \mathrm{0.634,0.689}\end{array}\right)$$
${\varvec{D}}{{\varvec{M}}}_{3}$	${C}_{1}$	${C}_{2}$	${C}_{3}$	${C}_{4}$	${C}_{5}$
${{\varvec{P}}}_{1}$	$$\left(\begin{array}{c}0.048, 0.676\\ \mathrm{0.450,0.676}\end{array}\right)$$	$$\left(\begin{array}{c}0.080, 0.677\\ \mathrm{0.448,0.677}\end{array}\right)$$	$$\left(\begin{array}{c}0.046, 0.685\\ \mathrm{0.164,0.685}\end{array}\right)$$	$$\left(\begin{array}{c}0.446, 0.679\\ \mathrm{0.446,0.679}\end{array}\right)$$	$$\left(\begin{array}{c}0.310, 0.689\\ \mathrm{0.634,0.689}\end{array}\right)$$
${{\varvec{P}}}_{2}$	$$\left(\begin{array}{c}0.323, 0.676\\ \mathrm{0.450,0.676}\end{array}\right)$$	$$\left(\begin{array}{c}0.119, 0.677\\ \mathrm{0.646,0.677}\end{array}\right)$$	$$\left(\begin{array}{c}0.021, 0.685\\ \mathrm{0.164,0.685}\end{array}\right)$$	$$\left(\begin{array}{c}0.664, 0.679\\ \mathrm{0.644,0.679}\end{array}\right)$$	$$\left(\begin{array}{c}0.046, 0.689\\ \mathrm{0.021,0.689}\end{array}\right)$$
${{\varvec{P}}}_{3}$	$$\left(\begin{array}{c}0.080, 0.676\\ \mathrm{0.234,0.676}\end{array}\right)$$	$$\left(\begin{array}{c}0.233, 0.677\\ \mathrm{0.119,0.677}\end{array}\right)$$	$$\left(\begin{array}{c}0.077, 0.685\\ \mathrm{0.228,0.685}\end{array}\right)$$	$$\left(\begin{array}{c}0.118, 0.679\\ \mathrm{0.644,0.679}\end{array}\right)$$	$$\left(\begin{array}{c}0.435, 0.689\\ \mathrm{0.076,0.689}\end{array}\right)$$
${{\varvec{P}}}_{4}$	$$\left(\begin{array}{c}0.048, 0.676\\ \mathrm{0.648,0.676}\end{array}\right)$$	$$\left(\begin{array}{c}0.322, 0.677\\ \mathrm{0.448,0.677}\end{array}\right)$$	$$\left(\begin{array}{c}0.440, 0.685\\ \mathrm{0.639,0.685}\end{array}\right)$$	$$\left(\begin{array}{c}0.644, 0.679\\ \mathrm{0.118,0.679}\end{array}\right)$$	$$\left(\begin{array}{c}0.310, 0.689\\ \mathrm{0.224,0.689}\end{array}\right)$$
${{\varvec{P}}}_{5}$	$$\left(\begin{array}{c}0.169, 0.676\\ \mathrm{0.120,0.676}\end{array}\right)$$	$$\left(\begin{array}{c}0.048, 0.677\\ \mathrm{0.646,0.677}\end{array}\right)$$	$$\left(\begin{array}{c}0.440, 0.685\\ \mathrm{0.639,0.685}\end{array}\right)$$	$$\left(\begin{array}{c}0.644, 0.679\\ \mathrm{0.118,0.679}\end{array}\right)$$	$$\left(\begin{array}{c}0.224, 0.689\\ \mathrm{0.310,0.689}\end{array}\right)$$

**Step-4(a)** Calculate the positive ideal solution and negative ideal solution for each DM in Table 17.

**Table 17:** $\mathrm{PIS}$ and $\mathrm{NIS}$ for each DM**.**

	${{\varvec{C}}}_{1}$	${{\varvec{C}}}_{2}$	${{\varvec{C}}}_{3}$	${{\varvec{C}}}_{4}$	${{\varvec{C}}}_{5}$
${\mathbf{P}\mathbf{I}\mathbf{S}}_{1}$	$$\left(\begin{array}{c}0.522, 0.676\\ \mathrm{0.234,0.676}\end{array}\right)$$	$$\left(\begin{array}{c}0.448, 0.677\\ \mathrm{0.080,0677}\end{array}\right)$$	$$\left(\begin{array}{c}0.639, 0.685\\ \mathrm{0.164,0.685}\end{array}\right)$$	$$\left(\begin{array}{c}0.644, 0.679\\ \mathrm{0.021,0.679}\end{array}\right)$$	$$\left(\begin{array}{c}\mathrm{0.634,0.689}\\ \mathrm{0.021,0.689}\end{array}\right)$$
${\mathbf{P}\mathbf{I}\mathbf{S}}_{2}$	$$\left(\begin{array}{c}0.323, 0.676\\ \mathrm{0.323,0.676}\end{array}\right)$$	$$\left(\begin{array}{c}0.646, 0.677\\ \mathrm{0.022,0677}\end{array}\right)$$	$$\left(\begin{array}{c}0.639, 0.685\\ \mathrm{0.440,0.685}\end{array}\right)$$	$$\left(\begin{array}{c}0.644, 0.679\\ \mathrm{0.021,0.679}\end{array}\right)$$	$$\left(\begin{array}{c}\mathrm{0.161,0.689}\\ \mathrm{0.224,0.689}\end{array}\right)$$
${\mathbf{P}\mathbf{I}\mathbf{S}}_{3}$	$$\left(\begin{array}{c}0.169, 0.676\\ \mathrm{0.120,0.676}\end{array}\right)$$	$$\left(\begin{array}{c}0.233, 0.677\\ \mathrm{0.119,0677}\end{array}\right)$$	$$\left(\begin{array}{c}0.440, 0.685\\ \mathrm{0.639,0.685}\end{array}\right)$$	$$\left(\begin{array}{c}0.644, 0.679\\ \mathrm{0.021,0.679}\end{array}\right)$$	$$\left(\begin{array}{c}\mathrm{0.435,0.689}\\ \mathrm{0.076,0.689}\end{array}\right)$$
${\mathbf{N}\mathbf{I}\mathbf{S}}_{1}$	$$\left(\begin{array}{c}0.080, 0.676\\ \mathrm{0.648,0.676}\end{array}\right)$$	$$\left(\begin{array}{c}048, 0.677\\ \mathrm{0.646,0677}\end{array}\right)$$	$$\left(\begin{array}{c}0.164, 0.685\\ \mathrm{0.440,0.685}\end{array}\right)$$	$$\left(\begin{array}{c}0.320, 0.679\\ \mathrm{0.644,0.679}\end{array}\right)$$	$$\left(\begin{array}{c}\mathrm{0.046,0.689}\\ \mathrm{0.637,0.689}\end{array}\right)$$
${\mathbf{N}\mathbf{I}\mathbf{S}}_{2}$	$$\left(\begin{array}{c}0.048, 0.676\\ \mathrm{0.649,0.676}\end{array}\right)$$	$$\left(\begin{array}{c}0.048, 0.677\\ \mathrm{0.646,0677}\end{array}\right)$$	$$\left(\begin{array}{c}0.077, 0.685\\ \mathrm{0.021,0.685}\end{array}\right)$$	$$\left(\begin{array}{c}0.048, 0.679\\ \mathrm{0.644,0.679}\end{array}\right)$$	$$\left(\begin{array}{c}\mathrm{0.114,0.689}\\ \mathrm{0.637,0.689}\end{array}\right)$$
${\mathbf{N}\mathbf{I}\mathbf{S}}_{3}$	$$\left(\begin{array}{c}0.048, 0.676\\ \mathrm{0.648,0.676}\end{array}\right)$$	$$\left(\begin{array}{c}0.048, 0.677\\ \mathrm{0.646,0677}\end{array}\right)$$	$$\left(\begin{array}{c}0.046, 0.685\\ \mathrm{0.164,0.685}\end{array}\right)$$	$$\left(\begin{array}{c}0.118, 0.679\\ \mathrm{0.118,0.679}\end{array}\right)$$	$$\left(\begin{array}{c}\mathrm{0.310,0.689}\\ \mathrm{0.634,0.689}\end{array}\right)$$

**Step-4(c)**: This step provides the distance measure among the NIS and PIS with each alternative. Distance measure from PIS and NIS of each alternative is given in Table 18.

**Table 18:** Distance measure from PIS and NIS of each alternative.

	${{\varvec{P}}}_{2}$	${{\varvec{P}}}_{2}$	${{\varvec{P}}}_{3}$	${{\varvec{P}}}_{4}$	${{\varvec{P}}}_{5}$
${\mathbf{D}\mathbf{I}\mathbf{S}}_{\mathbf{i}}^{+(1)}$	0.1018	0.2190	0.1941	0.1700	0.1029
${\mathbf{D}\mathbf{I}\mathbf{S}}_{\mathbf{i}}^{+(2)}$	0.0955	0.2290	0.1339	0.1675	0.1741
${\mathbf{D}\mathbf{I}\mathbf{S}}_{\mathbf{i}}^{+(3)}$	0.1927	0.1823	0.1631	0.0749	0.0828
${\mathbf{D}\mathbf{I}\mathbf{S}}_{\mathbf{i}}^{-(1)}$	0.2343	0.1013	0.1519	0.1541	0.2038
${\mathbf{D}\mathbf{I}\mathbf{S}}_{\mathbf{i}}^{-(2)}$	0.2175	0.0514	0.1709	0.1511	0.1537
${\mathbf{D}\mathbf{I}\mathbf{S}}_{\mathbf{i}}^{-(3)}$	0.0856	0.1933	0.1877	0.1592	0.1547
${\mathbf{D}\mathbf{I}\mathbf{S}}_{\mathbf{i}}^{-(3)}$	0.0856	0.1933	0.1877	0.1592	0.1547

**Step-4(d):** In this step, we find the relative closeness index using the equation of RCI. The RCI of each alternative is given in Table 19.

**Table 19:** Closeness for each DM.

${{\varvec{R}}{\varvec{C}}{\varvec{I}}}_{{\varvec{K}}}$	${{\varvec{P}}}_{1}$	${{\varvec{P}}}_{2}$	${{\varvec{P}}}_{3}$	${{\varvec{P}}}_{4}$	${{\varvec{P}}}_{5}$
${{\varvec{R}}{\varvec{C}}{\varvec{I}}}_{1}$	0.697	0.316	0.439	0.575	0.664
${{\varvec{R}}{\varvec{C}}{\varvec{I}}}_{2}$	0.695	0.183	0.561	0.474	0.469
${{\varvec{R}}{\varvec{C}}{\varvec{I}}}_{3}$	0.308	0.515	0.535	0.680	0.651

**Step-5:** In this step, we finalized the ranking of all EEG classier based on the score values**.** The score values of each EEG classifier and ranking are given in Table 20.

**Table 20:** Final closeness indices and Ranking of Alternatives.

Score of alternative						
Alternative	${{\varvec{P}}}_{1}$	${{\varvec{P}}}_{2}$	${{\varvec{P}}}_{3}$	${{\varvec{P}}}_{4}$	${{\varvec{P}}}_{5}$	Ranking
LDF-TOPSIS ((LDFWCA))	0.562	0.338	0.515	0.546	0.590	${{\varvec{P}}}_{5}>{{\varvec{P}}}_{1}>{{\varvec{P}}}_{4}>{{\varvec{P}}}_{3}>{{\varvec{P}}}_{2}$
LDF-TOPSIS (LDFWOCA)	0.462	0.398	0.415	0.4434	0.490	${{\varvec{P}}}_{5}>{{\varvec{P}}}_{1}>{{\varvec{P}}}_{4}>{{\varvec{P}}}_{3}>{{\varvec{P}}}_{2}$
LDF-TOPSIS (LDFWHCA)	0.582	0.477	0.487	0.546	0.619	${{\varvec{P}}}_{5}>{{\varvec{P}}}_{1}>{{\varvec{P}}}_{4}>{{\varvec{P}}}_{3}>{{\varvec{P}}}_{2}$

The ranking of all alternatives based on the Extended TOPSIS Method using LDFWCA, LDFWOCA, and LDFWHCA is given in Table 12. The overall ranking based on three proposed Copulas aggregation operators for LDFNs is ${{\varvec{P}}}_{5}>{{\varvec{P}}}_{1}>{{\varvec{P}}}_{4}>{{\varvec{P}}}_{3}>{{\varvec{P}}}_{2}$ and more the optimal selection of alternatives is ${{\varvec{P}}}_{5}$. This concludes that the proposed aggregation operators based on Copulas with an extended TOPSIS method are very appropriate for the GMCDM problem under the Linear Diophantine fuzzy information. In Table 12, the score values of each classifier are given and the score value of the Probabilistic neural network (PNN) is high compared with other EEG classifiers. The LDFNs represent information more extensive information than IFS PyFS and q-ROFS. In LDFNs, the membership and non-membership. In the decision-making process, the expert expresses his view of each alternative based on given some criteria under the Linear Diophantine fuzzy numbers and the LDFNS is more accurate and powerful to evaluate the alternative. The proposed new extended-TOPSIS method based on LDFWCA, LDFWOCA, and LDFWHCA is the more reliable and accurate result for ranking alternatives. The evaluation of the alternative process by the proposed method is more accurate and comprehensive as compared with other previous methods in Table 13.

## Comparative study

This section provides a comparison of the proposed method with some others operators and methods to fill the gap between existing approaches and also to show the validity and efficiency of the proposed methods. Previous research includes LDFW geometric aggregation (LDFWGA) operator by Raiz and Hashmi^23^, LDF Einstein aggregation operators developed by Lampan et. al,^25^, q-LDF weighted averaging and geometric aggregation operators presented by Almagrabi et.al^27^, and several more DM problems^22,24,26^. By solving the information data with some previous operators and coming to the same optimal decision, we equate our findings. The authors take different data given above and apply the proposed aggregation operators the results are the same as that of existing approaches shown in Table 21.

**Table 21:** Comparison Analysis of the Proposed Method with the Existence Method.

Score of Alternative						
Alternative	${{\varvec{P}}}_{1}$	${{\varvec{P}}}_{2}$	${{\varvec{P}}}_{3}$	${{\varvec{P}}}_{4}$	${{\varvec{P}}}_{5}$	Ranking
LDF-TOPSIS ((LDFWCA))	0.562	0.338	0.515	0.546	0.590	${{\varvec{P}}}_{5}>{{\varvec{P}}}_{1}>{{\varvec{P}}}_{4}>{{\varvec{P}}}_{3}>{{\varvec{P}}}_{2}$
LDF-TOPSIS (LDFWOCA)	0.462	0.398	0.415	0.434	0.490	${{\varvec{P}}}_{5}>{{\varvec{P}}}_{1}>{{\varvec{P}}}_{4}>{{\varvec{P}}}_{3}>{{\varvec{P}}}_{2}$
LDF-TOPSIS (LDFWHCA)	0.582	0.477	0.487	0.546	0.619	${{\varvec{P}}}_{5}>{{\varvec{P}}}_{1}>{{\varvec{P}}}_{4}>{{\varvec{P}}}_{3}>{{\varvec{P}}}_{2}$
LDFWA^23^	0.762	0.418	0.615	0.546	0.778	${{\varvec{P}}}_{5}>{{\varvec{P}}}_{1}>{{\varvec{P}}}_{3}>{{\varvec{P}}}_{4}>{{\varvec{P}}}_{2}$
LDFOWA^23^	0.622	0.517	0.597	0.525	0.687	${{\varvec{P}}}_{5}>{{\varvec{P}}}_{1}>{{\varvec{P}}}_{3}>{{\varvec{P}}}_{4}>{{\varvec{P}}}_{2}$
LDFHA^23^	0.562	0.498	0.515	0.534	0.690	${{\varvec{P}}}_{5}>{{\varvec{P}}}_{1}>{{\varvec{P}}}_{4}>{{\varvec{P}}}_{3}>{{\varvec{P}}}_{2}$
LDFEWA^25^	0.622	0.517	0.597	0.525	0.653	${{\varvec{P}}}_{5}>{{\varvec{P}}}_{1}>{{\varvec{P}}}_{3}>{{\varvec{P}}}_{4}>{{\varvec{P}}}_{2}$
LDFEOWA^25^	0.462	0.318	0.415	0.446	0.462	${{\varvec{P}}}_{5}>{{\varvec{P}}}_{1}>{{\varvec{P}}}_{4}>{{\varvec{P}}}_{3}>{{\varvec{P}}}_{2}$
LDFEHA^25^	0.502	0.446	0.327	0.477	0.519	${{\varvec{P}}}_{5}>{{\varvec{P}}}_{1}>{{\varvec{P}}}_{4}>{{\varvec{P}}}_{3}>{{\varvec{P}}}_{2}$
LDFWHG^25^	0.462	0.318	0.415	0.446	0.462	${{\varvec{P}}}_{5}>{{\varvec{P}}}_{1}>{{\varvec{P}}}_{4}>{{\varvec{P}}}_{3}>{{\varvec{P}}}_{2}$
q-LDFWA^27^	0.562	0.498	0.515	0.534	0.670	${{\varvec{P}}}_{5}>{{\varvec{P}}}_{1}>{{\varvec{P}}}_{4}>{{\varvec{P}}}_{3}>{{\varvec{P}}}_{2}$
q-LDFHA^27^	0.462	0.318	0.415	0.446	0.462	${{\varvec{P}}}_{5}>{{\varvec{P}}}_{1}>{{\varvec{P}}}_{4}>{{\varvec{P}}}_{3}>{{\varvec{P}}}_{2}$
q-LDFHA^27^	0.232	0.127	0.167	0.155	0.269	${{\varvec{P}}}_{5}>{{\varvec{P}}}_{1}>{{\varvec{P}}}_{3}>{{\varvec{P}}}_{4}>{{\varvec{P}}}_{2}$

In this study, we develop an intelligent based decision support system for classifier selection for EEG based on criteria under the linear diophantine fuzzy numbers. This study is free from statistical analysis, we use the fuzzy analysis method instead of statistical analysis. In this study, the proposed decision support systems analyzed five EEG classifiers for depression patients based on five types of entropy features, and the Probabilistic neural network (PNN) was chosen as the best EEG classifier. In Table 13, the score values of each classifier are given and the score value of the Probabilistic neural network (PNN) is high compared with other EEG classifiers. Even if the proposed CAD system is not used, the results apply to psychological exercise. Further examination of the data showed the information content of EEG signals of clinically depressed patients. The data of Table 1(a), 1(b), and 1(c) were analyzed by different techniques of decision support systems under the linear diophantine fuzzy numbers and these techniques proposed the best optimal EEG classier is Probabilistic neural network (PNN).

**Figure 2:**
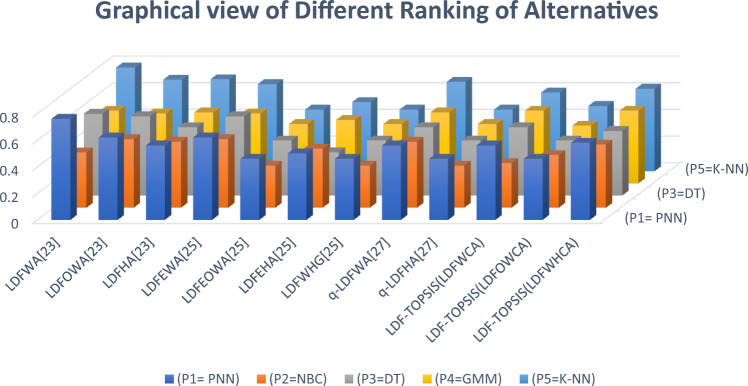
Graphical View of different ranking method results.

Figure 2 shows the values of alternative P 5, which is the best option based on the proposed method for decision-making under the various techniques.

With a classification rate of 99.5%, the classifier PNN performed significantly better than other classifiers in distinguishing between normal and depressed EEG signals in this paper. As a result, the proposed fuzzy decision support method can be used to diagnose and monitor depressed patients' treatment and care. When the above classifiers are used to analyze depressed patients and normal people, the proposed decision method produces accurate results.

### Limitation and discussion

In real life, hyperactivity disorder (HD), a neurodevelopmental disorder that affects a person's sleep, mood, anxiety, and learning, increases from time to time. Early diagnosis and timely medication can help people with HD perform everyday tasks without difficulty. EEG signals can help neuroscientists detect HD by examining changes that occur in them. EEG signals are complex, nonlinear and non-stationary. EEG signals are complex, nonlinear and unstable, which makes them difficult to detect visually, and existing DM^42,43^ models do not guarantee similar performance (unreliable). To handle this issue LDFNs represent information more extensive information than IFS PyFS and q-ROFS. In the decision-making process, the expert expresses his view of each alternative based on given some criteria under the Linear Diophantine fuzzy numbers and the LDFNS is more accurate and powerful to analyze the different EEG classifiers. The proposed new extended-TOPSIS method based on LDFWCA, LDFWOCA, and LDFWHCA is the more reliable and accurate result for analyzing the different EEG classifiers. The extended-TOPSIS method based on minimum distance to PIS and maximum distance to NIS plays a key role in dealing with this kind of practical problems. The proposed concept has a powerful ability to handle uncertainty in DM problems, but the theory has its limitations because most DM problems have two dimensions to handle uncertainty that the proposed method cannot handle. This proposed technique will enhance more by using the double hierarchy linguistic information instead of the real or fuzzy numbers.

## Conclusion

In a group decision or multi-expert decision-making (MEDM) problem, the decision expert needs an operator to aggregate the multi-expert opinion into single and collective decision information. To deal with this problem of aggregating the information of MEDM, different aggregation operators (AOs) were developed for aggregating the information. The most frequently used AOs in the MEDM are the weighted averaging (WA) aggregation operator and the weighted geometric (WG) aggregation operator. In this paper, the aggregation operators for LDFNs are obtained by using Copulas and Co-copulas. Furthermore, we developed some different Entropy measures and distance measures for the Extended-TOPSIS method for classifier selection based on some given criteria. For the data extraction of depression by using EEG, we need some future and optimal classifier selection by using different techniques. The main aim of this proposed work is to develop artificial intelligence-based fuzzy decision support systems (AI-FDSS) and intelligent decision support is an automatic decision process based on some input information. The AI-FDSS is considered for classifier selection for EEG under depression information based on given criteria. The proposed intelligent decision technique analyzes the k-nearest neighbor algorithm (k-NN), Gaussian mixture models (GMM), Decision tree (DT), Naïve Bayes classification (NBC), and Probabilistic neural network (PNN). The proposed technique is criterion based for analyzing optimal classifier selection for EEG under depression patients. First, we design a general algorithm for intelligent decision systems under the non-linear Diophantine fuzzy numbers to scrutinize the classifier selection technique under some different criteria. We use the classifier methods to obtain depression patients' data in normal situations and abnormal situations based on the given criteria. The proposed technique is criterion based for analyzing optimal classifier selection for EEG under depression patients. The proposed model for analyzing of classifier selection of EEG will compare with already existing models.

In the future, we will apply the Copulas and Copulas to Non-Linear Diophantine fuzzy numbers and will develop some different decision models, Also, Solve the Classifier selection through GRA, and VIKOR methods. We also develop some new aggregation operators for NLDFNs using the original work.

## Data Availability

There is no associate data with this research work and any one can use this data in his paper.
